# Cell cycle-arrested tumor cells exhibit increased sensitivity towards TRAIL-induced apoptosis

**DOI:** 10.1038/cddis.2013.179

**Published:** 2013-06-06

**Authors:** H Ehrhardt, F Wachter, M Grunert, I Jeremias

**Affiliations:** 1 Helmholtz Zentrum München, German Research Center for Environmental Health, Munich, Germany; 2 Division of Neonatology, Perinatal Center, University Children's Hospital, Ludwig-Maximilians-University Munich, Munich, Germany; 3 Department of Oncology/Hematology, Dr. von Haunersches Kinderspital, München, Germany

**Keywords:** cell cycle arrest, TRAIL, apoptosis, DR5, cyclins

## Abstract

Resting tumor cells represent a huge challenge during anticancer therapy due to their increased treatment resistance. TNF-related apoptosis-inducing ligand (TRAIL) is a putative future anticancer drug, currently in phases I and II clinical studies. We recently showed that TRAIL is able to target leukemia stem cell surrogates. Here, we tested the ability of TRAIL to target cell cycle-arrested tumor cells. Cell cycle arrest was induced in tumor cell lines and xenografted tumor cells in G0, G1 or G2 using cytotoxic drugs, phase-specific inhibitors or RNA interference against cyclinB and E. Biochemical or molecular arrest at any point of the cell cycle increased TRAIL-induced apoptosis. Accordingly, when cell cycle arrest was disabled by addition of caffeine, the antitumor activity of TRAIL was reduced. Most important for clinical translation, tumor cells from three children with B precursor or T cell acute lymphoblastic leukemia showed increased TRAIL-induced apoptosis upon knockdown of either cyclinB or cyclinE, arresting the cell cycle in G2 or G1, respectively. Taken together and in contrast to most conventional cytotoxic drugs, TRAIL exerts enhanced antitumor activity against cell cycle-arrested tumor cells. Therefore, TRAIL might represent an interesting drug to treat static-tumor disease, for example, during minimal residual disease.

Resting tumor cells exhibit a severe challenge during anticancer treatment. In minimal residual disease, surviving tumor cells might stay quiescent and induce relapse after a prolonged period of time. Resting tumor cells display enhanced treatment resistance compared with actively cycling tumor cells, as several groups of anticancer drugs directly target the active cell cycle. Cancer stem cells are known to remain resting. To increase the prognosis and cure rate of cancer, anticancer therapy has to remove resting tumor cells.^1, 2, 3, 4^

TNF-related apoptosis-inducing ligand (TRAIL) is a promising future anticancer drug due to its tumor selectivity, almost in the absence of side effects in animal trials, and phases I and II clinical studies.^5, 6^ The intracellular apoptosis signal transduction initiated by TRAIL is well characterized and involves the TRAIL-death receptors, FADD (Fas-associated protein with death domain) as adapter protein and caspases. The apoptosis signal of TRAIL might be amplified by mitochondria, which is regulated by members of the Bcl-2 family. Further regulators of TRAIL-induced apoptosis are the Caspase-8 antagonist FLIP (FLICE inhibitory protein) and members of the IAP-family including XIAP, which antagonize downstream caspases.^7, 8, 9^

Although the antitumor effect of TRAIL as a single agent is limited, TRAIL exerts remarkable antitumor activity upon combination with established cytotoxic drugs in phases I and II clinical trials. Cytotoxic drugs like doxorubicin (doxo) or methotrexate (MTX) and others induce synergistic apoptosis upon combination with TRAIL.^7, 8, 10, 11, 12, 13, 14^

Several different mechanisms have been described of how cytotoxic drugs sensitize tumor cells towards TRAIL-induced apoptosis. Among them, the transcription factor p53 is activated by several established cytotoxic drugs and mediates a number of different effects in tumor cells including gene regulation, apoptosis and cell cycle arrest.^15, 16^ As several proteins mediating or regulating TRAIL-induced apoptosis are p53 target genes, for example, TRAIL receptor 2 (death receptor 5, DR5), p53-mediated gene regulation is suggested to be the main mechanism for mediating synergistic apoptosis of cytotoxic drugs and TRAIL.^6, 9, 14, 17^

We have recently described the importance of p53-mediated cell cycle arrest for inhibiting vinca alkaloid-induced apoptosis.^18^ We also described that TRAIL damages stem cell surrogates in patient-derived leukemia cells.^19^ As cancer stem cells are often resting, we hypothesized that TRAIL might be able to induce apoptosis in resting tumor cells. Although chemical compounds or drugs in preclinical testing were shown to sensitize tumor cells towards TRAIL-induced apoptosis accompanied by cell cycle arrest,^20, 21, 22^ no molecular data exist so far and no data on patients' tumor cells are present. Therefore, we studied here how cell cycle arrest influences the ability of TRAIL to induce apoptosis in tumor cells, using molecular approaches in patient-derived tumor cells.

## Results

### Several cytotoxic drugs sensitize towards TRAIL-induced apoptosis and induce p53-typic effects

Numerous conventional cytotoxic drugs of current clinical routine are known to induce cell cycle arrest mediated by the transcription factor p53. In a first approach, cell cycle arrest was induced using cytotoxic drugs.

**Figure 1:**
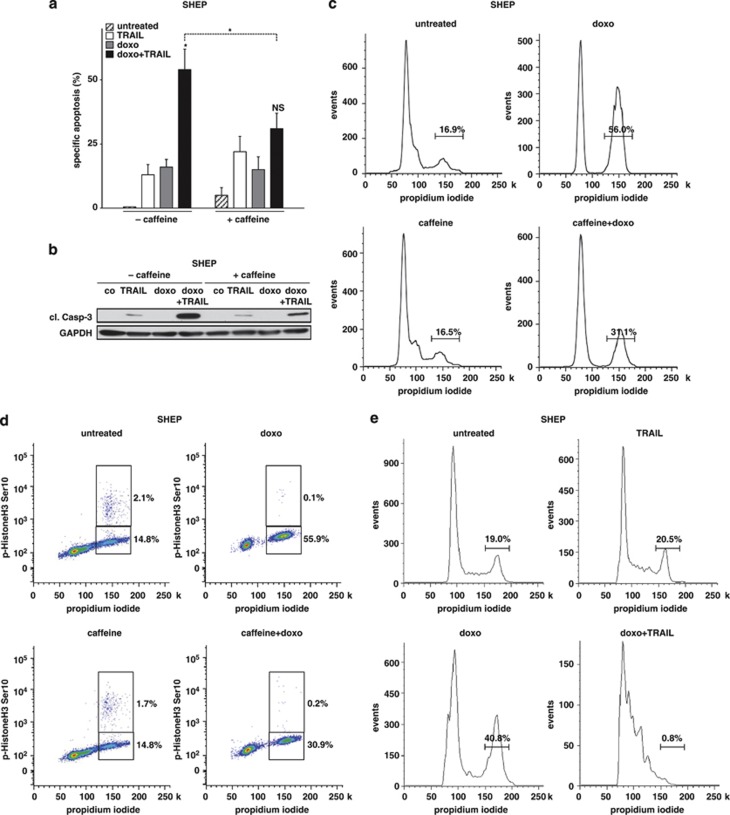
Super-additive activity of doxo and TRAIL in SHEP cells. (**a**) SHEP cells were pretreated with caffeine (300 *μ*g/ml) for 12 h or left untreated, followed by stimulation with doxo (100 ng/ml) for 48 h. TRAIL (100 ng/ml) was added afterwards for another 24 h. (**b**) SHEP cells pretreated and stimulated as in(a) were analyzed for caspase-3 cleavage. co=untreated, cl. Casp-3=cleaved Caspase-3. (**c**, **d**) SHEP cells treated with caffeine and doxo as in (a) were analyzed for cell cycle distribution using propidium iodide staining (**c**). G2 and M-phase were discriminated by the simultaneous staining for p-HistoneH3Ser10 (**d**). (**e**) SHEP cells were stimulated with doxo (30 ng/ml) for 48 h, followed by TRAIL (10 ng/ml). Cell cycle analysis was performed 24 h after the addition of TRAIL using propidium iodide staining, as in (**c**), gating on living cells. Cell death induction was measured by Nicoletti staining. Statistical analysis was performed comparing the apoptosis induction of the combined stimulation to the addition of cell death induction by doxo and TRAIL alone, and comparing the combinatorial application of pretreated and untreated cells with paired *t*-test. ^*^P<0,05, NS=statistically not significant

**Figure 2:**
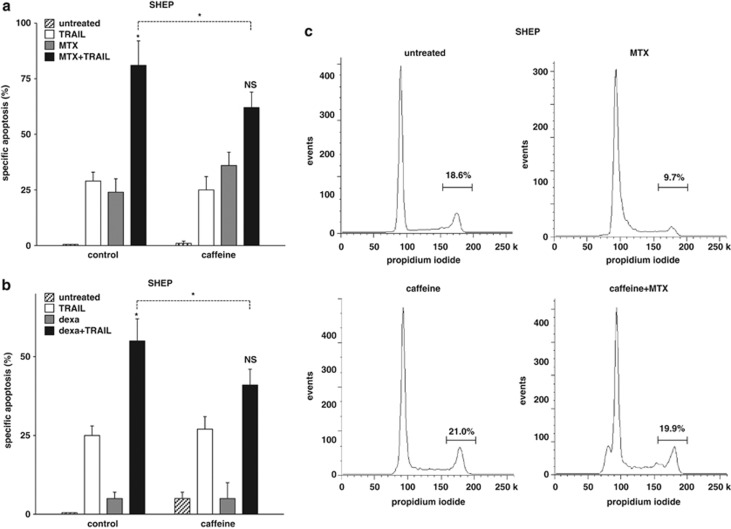
Augmented apoptosis-inducing capacity of TRAIL associated with cell cycle arrest by MTX and dexamethasone. (**a**, **b**) SHEP cells were pretreated with caffeine for 12 h, followed by stimulation with methotrexate (30 *μ*M; (**a**) or dexamethasone (10^−5 ^M; (**b**) for 48 h. TRAIL (100 ng/ml) was added afterwards for another 24 h. (c) SHEP cells treated with caffeine and MTX ,as in (a), were analyzed, as in Figure 1b. Determination of apoptosis induction, presentation and analysis of the data and statistical analysis were performed as in Figure 1. *P<0.05

**Figure 3:**
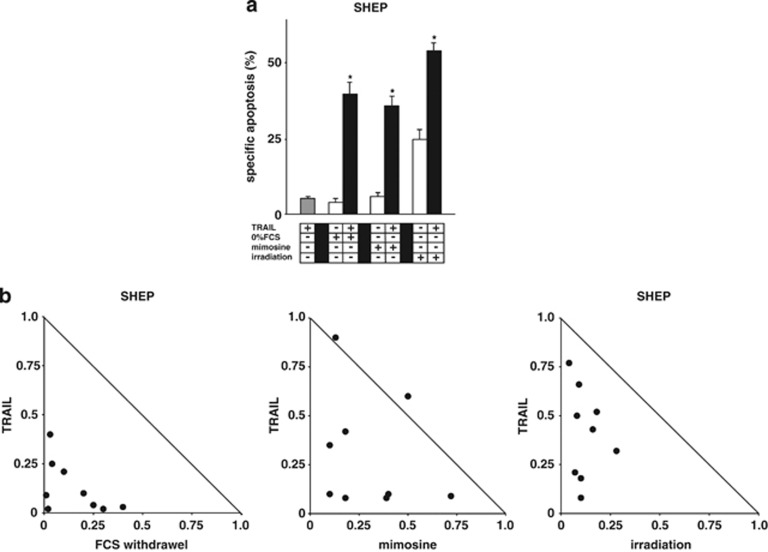
Biochemical cell cycle arrest sensitizes for TRAIL. (**a**) SHEP cells were pretreated with FCS withdrawal, preincubated with mimosine (100 *μ*M) or irradiated with 30 Gy for 24 h. TRAIL (100 ng/ml) was added for another 24 h. (**b**) Isobolograms were applied to the experimental setting from (**a**) to test for synergistic effects of FCS withdrawal (0, 0,1 and 1% FCS), mimosine (10, 30 and 100 *μ*M) treatment or irradiation (3, 6 and 10 Gy) plus TRAIL (3, 10 and 30 ng/ml). Determination of apoptosis induction, presentation and analysis of the data, and statistical analysis were performed as in Figure 1. ^•^Each data point represents the dose-equation of the applied combinatorial treatment. *P<0.05

**Figure 4:**
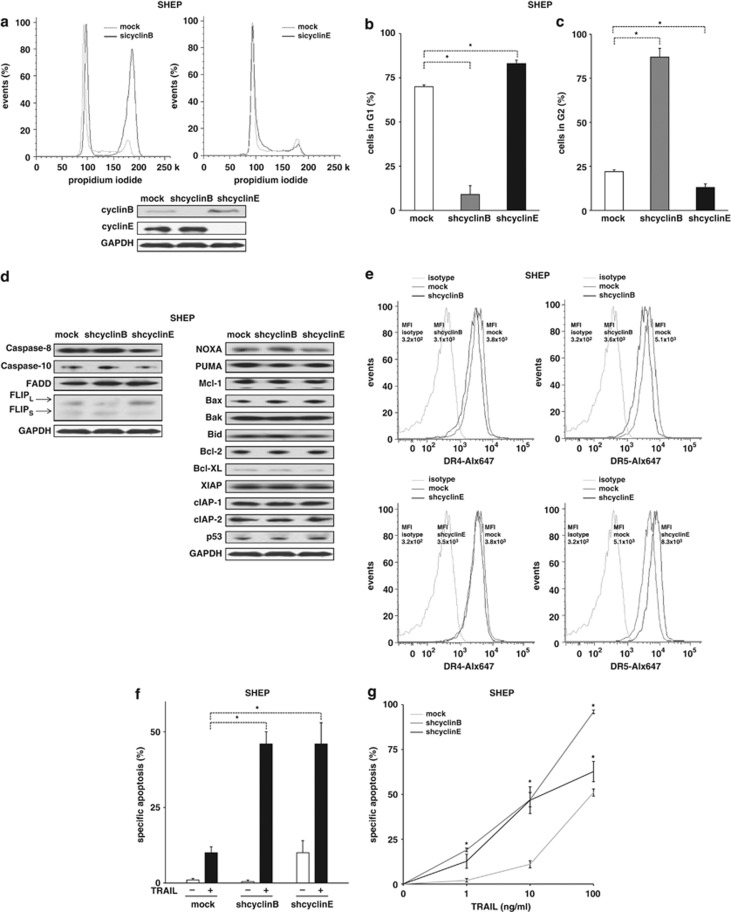
Molecular cell cycle arrest sensitizes for TRAIL. (**a**) SHEP cells transfected with shRNA against cyclinB or cyclinE were analyzed for the cell cycle distribution, as in Figure 1c. (**b**–**e**) SHEP cells from (**a**) were analyzed for cell cycle distribution in G1 (**b**) and G2 (**c**), apoptosis protein expression (**d**) and TRAIL-death receptor-1 and -2 expression (**e**) using the experimental setting from Figures 1c and d and Supplementary Figures 1A and 1B. (**f**) SHEP cells from (**a**) were stimulated with TRAIL (10 ng/ml). (**g**) TRAIL dose–response curves were performed in parental SHEP cells, and cells with knockdown of cyclinB or cyclinE from (**a**). Determination of cell cycle distribution and apoptosis induction, presentation and analysis of the data, and statistical analysis were performed as in Figure 1. For multivariate analysis, RM ANOVA was used. *P<0.05

**Figure 5:**
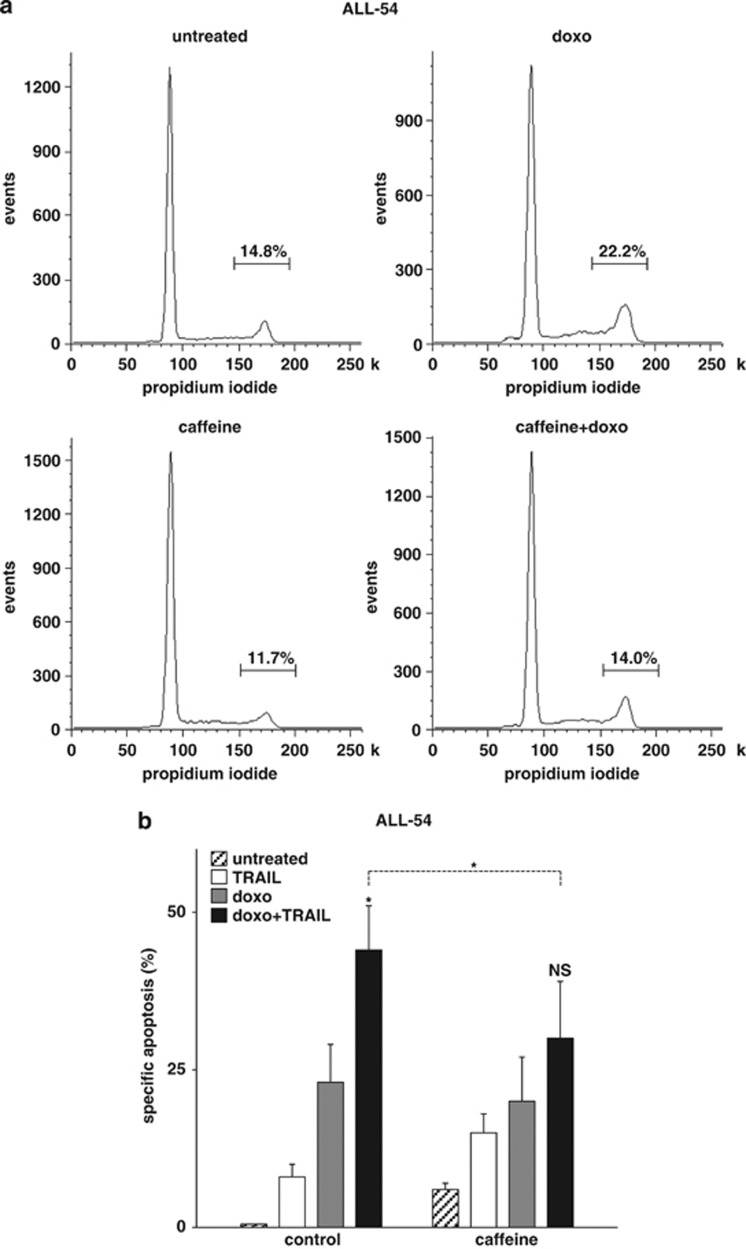
Doxo-induced cell cycle arrest associated with efficient TRAIL apoptosis induction in xenografted ALL cells. (**a**, **b**) Xenografted pre-B ALL-54 cells were pretreated with caffeine (100 *μ*g/ml) for 12 h, followed by stimulation with doxo (30 ng/ml). After 24 h of incubation with doxo, cell cycle analysis was performed (**a**) or cells were stimulated with TRAIL (10 ng/ml) for another 24 h (**b**). Determination of cell cycle distribution and apoptosis induction, presentation and analysis of the data and statistical analysis were performed, as in Figure 1. *P<0.05

**Figure 6:**
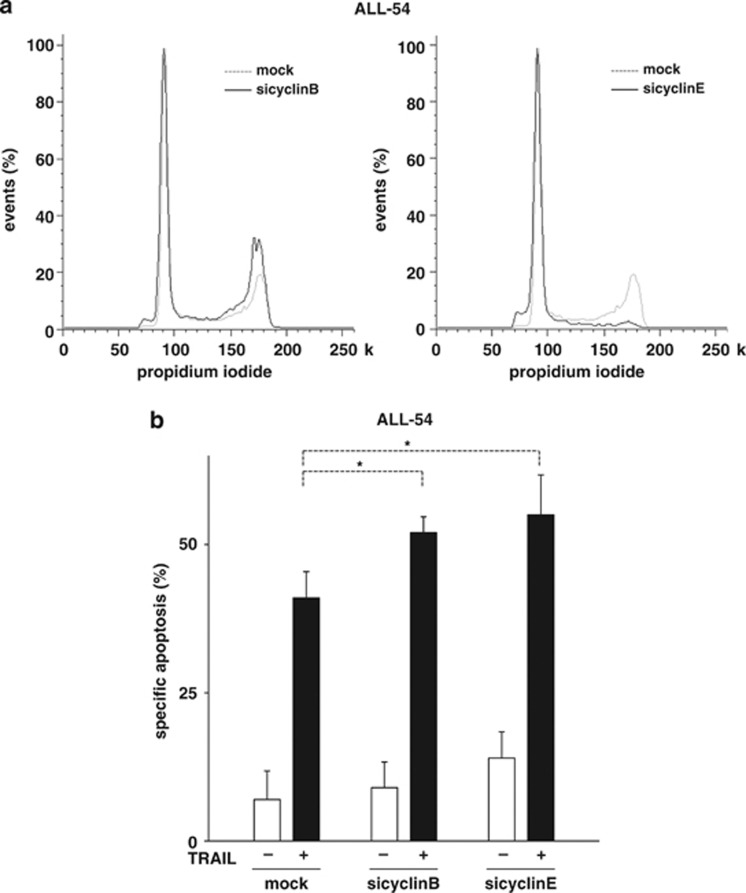
Molecular cell cycle arrest in G1 or G2 promotes apoptosis induction by TRAIL in xenografted ALL cells. (**a**, **b**) ALL-54 cells were transiently transfected with siRNA against cyclinB, cyclinE or a mock sequence using single nucleofection, as described in Materials and Methods. Cells were investigated for cell cycle distribution 24 h after transfection (**a**) or were stimulated with TRAIL (10 ng/ml) for another 24 h (**b**). Determination of cell cycle distribution and apoptosis induction, presentation and analysis of the data, and statistical analysis were performed as in Figures 1 and 4. *P<0.05

We used SHEP neuroblastoma cells (printed Figures 1, 2, 3, 4) and HCT116 colon cancer cells (Supplementary Figures S2, S4 and S5), both of which express functionally active p53 in wild-type conformation.^23^ Additionally, CEM T-ALL leukemia cells were included with mutant, but functionally active, p53 (Supplementary Figure S6)^11, 18, 23, 24^ and xenografted ALL leukemia samples (Figures 5 and 6 and Supplementary Figure S7). As described for several cell lines in the literature,^12, 13, 14^ both SHEP and HCT116 cells displayed prominent synergistic apoptosis induction when TRAIL was combined with doxo (Figure 1a and Supplementary Figure S2A). Apoptosis data were congruent with increased caspase cleavage for the drug combination (Figure 1b). Concomitantly, doxo strongly activated p53 in both cell lines. According to the known different effects of p53, doxo upregulated typical p53 target genes in the TRAIL apoptosis signaling pathway, mainly TRAIL receptor-2 and Caspase-10, and arrested the cell cycle (Figures 1c and d, Supplementary Figures S1A and B, and S2B and C). The upregulation of TRAIL receptor-2 by doxo was higher in cells with cell cycle arrest in G2 (Supplementary Figure S1C). As TRAIL receptor-2 regulation has been reported to be a central determinant of TRAIL sensitivity, overexpression of TRAIL receptor-2 was performed, but did not have an impact on the TRAIL response, arguing against a dominant role of TRAIL receptor-2 expression levels in the regulation of TRAIL sensitivity in our experimental setting (Supplementary Figure S1D). Determination of the phosphorylation status at Serine 10 of Histone H3 revealed that the cell cycle was arrested in G2, but not in M (Figures 1c and d, Supplementary Figures S2D and E). For the combination of doxo plus TRAIL, the fraction of cells in G2 was markedly reduced (Figure 1e). To prevent the cell cycle arrest by cytotoxic drugs, the biochemical inhibitor caffeine was used, as it is highly efficient with nearly absent toxicity and not specific to a certain phase of the cell cycle or chemotherapeutic drug applied. Synergistic apoptosis induction was markedly reduced by pretreatment with caffeine, which also prevented p53 accumulation, reduced the upregulation of TRAIL receptor-2 and Caspase-10, and the cell cycle arrest by doxo (Figures 1a–d, Supplementary Figures S1A and B, and S2A-E).

Similarly, MTX and dexamethasone (dexa) induced super-additive apoptosis with TRAIL (Figures 2a and b), and both drugs arrested the cell cycle in G1 (Figure 2c and data not shown). Whereas MTX increased the expression of TRAIL receptor-2, dexa did not relevantly alter the mean fluorescence intensity (Supplementary Figure S3). Pretreatment with caffeine reduced synergistic apoptosis, TRAIL receptor-2 upregulation and cell cycle arrest (Figures 2a–c, Supplementary Figure S3 and data not shown).

Taken together, several cytotoxic drugs sensitized towards TRAIL-induced apoptosis, induced cell cycle arrest at different phases and upregulated typical p53 target genes, which were all inhibited by caffeine.

### Cell cycle inhibitors sensitize for TRAIL-induced apoptosis

Cytotoxic drugs are mainly described to promote TRAIL-induced apoptosis by the regulation of apoptosis protein expression including TRAIL receptor-2 expression.^6, 9, 14, 15, 16, 17^ The data presented so far do not allow estimation of the specific contribution of cell cycle arrest. Next, we aimed at discriminating between the different effects of p53 induced by cytotoxic drugs, and asked whether cell cycle arrest itself might be sufficient to sensitize towards TRAIL-induced apoptosis. Towards this aim, we used biochemical cell cycle inhibitors or irradiation, which are known to arrest cells in defined phases of the cell cycle. As published before, FCS withdrawal arrested the cells in G0, mimosine in G1 and irradiation in G2 (Table 1 and Supplementary Table 1).^25^ Interestingly, upon arresting the cell cycle in any given phase, both compounds and irradiation significantly sensitized for TRAIL-induced apoptosis (Figure 3a). The analysis with isobolograms showed the synergistic effects clearly (Figure 3b and Supplementary Figure S4). Taken together, biochemical cell cycle arrest was associated with particularly efficient cell death induction by TRAIL.

### Knockdown of cyclinB or cyclinE induce cell cycle arrest and sensitize for TRAIL-induced apoptosis

So far, we showed that various drugs, compounds and stimuli induced cell cycle arrest and sensitized towards TRAIL-induced apoptosis. We next asked whether cell cycle arrest was mechanistically responsible for sensitizing towards TRAIL-induced apoptosis. To discriminate between drug-induced cell cycle arrest and further drug-induced, p53-mediated effects such as gene regulation, cell cycle arrest was induced by molecular manipulation using RNA interference.

Cyclins are regulators of the cell cycle that control transition through the different phases of the cell cycle. Whereas expression of cyclinB is a prerequisite for transition from G2 to M, cyclinE controls the slip from G1 to S-phase. Using RNA interference, we studied the impact of downregulation of these cyclines on TRAIL-induced apoptosis.

According to published data, knockdown of cyclinB or cyclinE induced cell cycle arrest in G2 or G1 (Figures 4a–c and Supplementary Figure S5A). As published for the cell cycle arrest in G1 by inhibition of cyclinD1 before, we preferred an siRNA sequence against cyclinE that leads to an incomplete, but statistically significant cell cycle arrest in G1, but does not affect the basal apoptosis rate of transfected cells.^25, 26, 27^ The expression levels of the apoptosis signaling proteins studied and p53 remained unchanged in cells with knockdown of cyclinB or cyclinE. Of special interest, the expression level of TRAIL receptor-1 and -2 remained unchanged (Figures 4d and e). Knockdown of cyclinB or cyclinE significantly sensitized the solid tumor cell lines SHEP and HCT116 for apoptosis induction by TRAIL (Figure 4f and Supplementary Figure S5B). In line with our previous results for vincristine-induced apoptosis, the slight alteration of cells in G1 was associated with a marked difference in apoptosis induction by TRAIL.^25^ Dose–response curves confirmed the general impact of knockdown of cyclinB or E on TRAIL-induced apoptosis (Figure 4g).

To prove the general significance of the observed phenotype across different tumor entities, the hematopoietic T cell leukemia cell line CEM was additionally studied, which expresses mutant but functionally active p53.^11, 18, 23, 24, 28^ siRNA against cyclinB and E arrested the cell cycle in G2 and G1, respectively, also to a minor extent compared with the solid tumor cell lines (Supplementary Figure S6A). Similar to the solid tumor cell lines studied, cell cycle arrest sensitized towards TRAIL-induced apoptosis in CEM cells (Supplementary Figure S6B).

These data show that cell cycle arrest itself sensitizes towards TRAIL-induced apoptosis in the clear absence of protein regulation.

### Patient-derived tumor cells are sensitized for TRAIL-induced apoptosis by cytotoxic drugs

Established cell lines might have acquired additional, non-physiologic mutations upon prolonged culture *in vitro*. For example, the majority of leukemic cell lines inherit mutations in p53, which are rarely found in leukemia patients.^23, 29, 30, 31^ To exclude a culture-specific artifact and to go beyond cell line work, tumor cells from patients were studied. Towards this aim, we used primary tumor cells from children with acute leukemia. As these cells are notoriously reluctant towards *in vitro* growth, primary cells were passaged through immunocompromised mice,^11, 32^ where they remain largely genetically stable.^33^

Three different ALL samples were stimulated with doxo and TRAIL, with and without pretreatment with caffeine. Whereas doxo partially arrested the cells in G2, caffeine markedly reduced the G2 arrest (Figure 5a and Supplementary Figure S7A). On a functional level and in accordance to data obtained in cell lines, doxo and TRAIL induced synergistic apoptosis, which was inhibited by pretreatment with caffeine (Figure 5b and Supplementary Figures S7B and C).

### Patient-derived tumor cells are sensitized towards TRAIL-induced apoptosis by knockdown of cyclinB or cyclinE

To prove that cell cycle arrest was capable to sensitize towards TRAIL-induced apoptosis, patient-derived ALL cells were transfected with siRNA targeting cyclinB or E, using our recently described technique.^11, 24, 32^ Whereas siRNA against cyclinB accumulated cells in G2, siRNA against cyclinE increased the fraction of cells in G1 (Figure 6a and data not shown). Concomitantly, knockdown of either cyclinB or cyclinE augmented TRAIL-induced apoptosis in ALL cells of all three patients (Figure 6b and Supplementary Figures S7D and E).

Thus, cell cycle arrest augmented TRAIL-induced apoptosis not only in cell line cells, but also in tumor cells derived from various children with B precursor ALL. Taken together and in contrast to conventional chemotherapeutics, TRAIL induces apoptosis more efficiently in tumor cells during cell cycle arrest compared with actively cycling tumor cells.

## Discussion

Our data show that TRAIL induces apoptosis more efficiently if tumor cells undergo cell cycle arrest compared with actively cycling tumor cells. For the first time, we obtained mechanistic proof that cell cycle arrest itself sensitizes tumor cells towards TRAIL-induced apoptosis, including patients' tumor cells. This finding was obtained by inducing cell cycle arrest by (i) conventional cytotoxic drugs; (ii) known cell cycle arrestors or (iii) molecularly by knockdown of certain cyclines. Knockdown-induced cell cycle arrest sensitized towards TRAIL-induced apoptosis in cell lines of various different tumor entities, as well as in patient-derived leukemia cells.

Therapeutic targeting of cells in cell cycle arrest is of high clinical importance. Cancer stem cells are known for their low cycling activity and chemoresistance. Static-tumor diseases are especially difficult to treat, for example, during minimal residual disease or in low-grade tumors. Insufficient treatment of static-tumor disease often results in tumor relapse. Our finding might suggest testing TRAIL in static-tumor disease *in vivo* as TRAIL seems to be especially efficient against resting tumor cells.

As TRAIL induces limited apoptosis in most primary tumor cells when given alone, the combined use of TRAIL together with conventional cytotoxic drugs has been intensively studied over the last years. Several different conventional anticancer drugs strongly sensitize tumor cells towards TRAIL-induced apoptosis. In search for underlying signaling mechanisms, p53 and its downstream effects were studied intensively. Most cytotoxic drugs accumulate and activate p53. p53-mediated gene regulation of signaling mediators of TRAIL-induced apoptosis such as TRAIL receptor-2 was thought to be responsible for drug-induced sensitization towards TRAIL-induced apoptosis. These considerations were used to optimize combinatorial approaches involving TRAIL.^6, 8, 9, 14, 17, 34^

Besides protein regulations, p53 induces cell cycle arrest. Although p53 is mutated in many tumor cells, leading to altered p53 function, induction of cell cycle arrest is not affected by loss of DNA-binding capacity in most p53 mutants.^34, 35^ Our data show that in addition to the dominant p53-mediated gene regulation, p53-mediated cell cycle arrest represents a mechanism by which cytotoxic drugs sensitize tumor cells towards TRAIL-induced apoptosis mediated by p53.

We have recently described that anthracyclines and vinca alkaloids are less effective when applied simultaneously as anthracyclines induce cell cycle arrest, whereas vinca alkaloids require active cell cycling for antitumor efficiency.^18^ In contrast, cell cycle arrest is beneficial for TRAIL. The data presented here widen the therapeutic potential for TRAIL to all phases of the cell cycle. Our data add to the controversial discussion, whether or when cell cycle arrest is beneficial, irrelevant or detrimental during anticancer therapy, for example, using TRAIL.^18, 20, 21, 22, 34, 35, 36, 37, 38^

Increased activity of TRAIL against resting tumor cells might explain why TRAIL is especially effective in combination with cytostatic drugs, which induce cell cycle arrest. Our data highlight TRAIL as a promising candidate in contrast to others for the combination with cytostatic drugs.^18, 20, 37, 39^ Future polychemotherapy protocols might position TRAIL in close relation to cell cycle in order to gain highest antitumor efficiency by TRAIL, based on the molecular understanding of drug–drug interactions.

## Materials and Methods

### Materials

TRAIL was obtained from Pepro Tech (Hamburg, Germany). Alternatively, trimerized TRAIL was produced as described recently, rendering identical results.^39^ Caffeine and L-mimosine were obtained from Calbiochem (Darmstadt, Germany); all further reagents were obtained from Sigma (St. Louis, MO, USA).

For flow cytometric analysis, the following antibodies were used: anti-cyclinD1 from BD Biosciences (San Jose, SA, USA) and anti-p-HistoneH3Ser10 from Cell Signaling Technology (Danvers, MA, USA), and anti-DR4 and anti-DR5 from Alexis Corp. (Lausen, Switzerland), Alx647-conjugated secondary anti-mouse antibody was obtained from Invitrogen (Darmstadt, Germany); for western blot: anti-FADD, anti-FLIP and anti-XIAP from BD Biosciences, anti-Bcl-xL, anti-Bid, anti-cIAP-1 and anti-PUMA from Cell Signaling; anti-Bak, anti-Bax, anti-Bcl-2, anti-cIAP-2 and anti-p53 from Santa Cruz (Santa Cruz, CA, USA); anti-Caspase-10 from MBL International (Woburn, MA, USA); anti-GAPDH from Thermo Fisher (Waltham, MA, USA); anti-NOXA from Calbiochem (San Diego, CA, USA), and anti BIM and anti Caspase-8 from Alexis Corp.

### Cell lines, xenograft ALL cells and transfection experiments

HCT116 p53 +/+ were obtained from B. Vogelstein (The Johns Hopkins University School of Medicine, Baltimore, MD, USA). All further cell lines were obtained from DSMZ (Braunschweig, Germany). For leukemic cell line experiments, cells were seeded at 0.25 × 10^6^/ml for stimulations of solid tumor cells at 0.05 × 10^6^/ml. Tumor cells were incubated with caffeine and chemotherapeutic drugs, as indicated in the corresponding figure legends.

Informed consent was obtained from all patients in written form, and studies were approved by the ethical committee of the medical faculty of the Ludwig Maximilians University Munich (LMU 068-08) and the Children's Hospital of the TU Munich (TU 2115/08). Animal work was approved by the Regierung von Oberbayern (55.2-1-54-2531-2-07). The xenograft mouse model, patient characteristics and engraftment, amplification, isolation and standardized procedures of *in vitro* stimulation have been described in detail recently.^11, 25, 32^

Transfection experiments in HCT116 was performed with lipofectamine 2000 (Life Technologies, Grand Island, NJ, USA) according to the manufacturers' instructions. Lentiviral transduction of SHEP cells was described recently.^18, 24, 28^ Nucleofection of CEM cells was performed with one million cells per reaction, and of xenograft ALL cells with five million cells per reaction using Amaxa Nucleofector technology (Lonza, Cologne, Germany) and program C16.^32^ Lipofection in HCT116 and nucleofection in CEM cells was performed three times consecutively every 12 h. Lentiviral transduction was performed annealing the following sense and corresponding antisense oligonucleotides for the generation of pGreen-Puro shcyclinB 5′-GTCGGATCCGAAATGTACCCTCCAGAAATTGAATTCGTTTCTGGAGGGTACATTTCTTTTTAAGCTTAGT-3′ and 5′-GATCCAAGTGCTACTGCCGCAGTATCTTCA AGAGAGATACTGCGGCAGTAGCACTTTTTTTC-3′ for the construct containing shcyclinE.^24^ DR4 and DR5 cDNAs were obtained from imaGENES GmbH (Berlin, Germany) and were cloned into pcDNA3.1.^39^ For the transient knockdown of cyclinB or cyclinE in HCT116, CEM and xenograft ALL cells, siRNA against cyclinB (5′-GAAAUGUACCCUCCAGAAAtt-3′, 20 *μ*M) and siRNA against cyclinE 5′-AAGTGCTACTGCCGCAGTATCtt-3′, 20 *μ*M) were obtained from MWG Biotech (Ebersberg, Germany). All Star negative control siRNA from Qiagen (Hilden, Germany) was used for control transfection.

### Apoptosis assays, flow cytometric analyses and western blot

Determination of cell death induction was performed with forward side scatter analyses for leukemia cells and with Nicoletti staining for solid tumor cells. Apoptotic cell death was confirmed in selected experiments with Annexin V–propidium iodide double staining as described.^11, 18, 28^ To discriminate between G2 and M arrest, double staining with p-Histone H3 and propidium iodide was performed; to separate G0 and G1 phase, double staining with cyclinD1 and propidium iodide was performed, as described recently.^18, 25^ After cell fixation in 70% ethanol overnight, cells were resuspended in PBS with 0.25% Triton X, followed by incubation with the specific antibodies overnight in PBS supplemented with 1% BSA. After three washing steps, cells were incubated with RNAse A (100 *μ*g/ml) at 37 °C and propidium iodide was added at 10 *μ*g/ml directly before flow cytometric analysis. For the detection of TRAIL-death receptors, cells were incubated with the specific primary antibody, followed by incubation with an anti-mouse IgG1-specific antibody conjugated to Alx647 (Life Technologies). To exclude dead cells, death receptor staining was followed by Annexin V (BD Biosciences)/propidium iodide (1 *μ*g/ml) double staining. LSR II (BD Biosciences) was used for the determination of cell cycle distribution and TRAIL receptor expression gating on living cells, and data were analyzed using Flow Jo software version 8.8.6 (Ashland, OR, USA). Western blot analysis was performed using a lysis buffer containing 20 mM Tris-HCl (pH 7.5), 150 mM NaCl, 1 mM Na_2_ EDTA, 1 mM EGTA, 1% Triton X, 2.5 mM sodium pyrophosphate, 1 mM beta-glycerophosphate and 1 mM Na_3_VO_4_ supplemented with proteinase inhibitor cocktail set I (Merck, Darmstadt, Germany) according to the manufacturer's instructions.

### Statistical analysis

Specific apoptosis was calculated as ((apoptosis of stimulated cells at end minus apoptosis of unstimulated cells at end) divided by (100 minus apoptosis of unstimulated cells at end) times 100).

All data are presented as the mean values of at least three independent experiments±S.E.M., unless otherwise stated. Isobolograms were performed with CompuSyn software version 1.0 (ComboSyn Inc., Paramus, NJ, USA). To test for significant differences, the paired *t*-test was applied to compare two groups; for multivariate analysis, one-way RM ANOVA was used. Statistical significance was accepted with *P*<0.05.

## Figures and Tables

**Table 1:** Cell cycle distribution in SHEP cells after cell cycle inhibition

*Cell cycle distribution*	*G0 (*%)	*G1 (*%)	*G2 (*%)	*M (*%)
Control	0.2±0.2	67.8±3.6	18.8±4.5	2.8±0.8
0% FCS	30.9±0.9	52.6±2.9	9.8±0.5	0.2±0.1
Mimosine	0.1±0.1	85.0±3.5	10.9±1.0	0.1±0.1
Irradiation	1.1±0.3	15.8±2.3	75.2±3.8	0

SHEP cells were treated by withdrawal of FCS (0% FCS), with mimosine (100 *μ*M) or irradiated (30 Gy) for 24 h. Cell cycle distribution was analyzed with propidium iodide in combination with cyclinD1 staining to discriminate G0 and G1 phases, and with p-Histone H3 staining to separate the arrest in G2 and M-phase. Data are presented as mean±S.E.M. of three independent experiments.

## Abbreviations

ALL: acute lymphatic leukemia
Bcl-2: B-cell lymphoma 2
Dexa: Dexamethasone
Doxo: Doxorubicin
FADD: Fas-associated protein with death domain
FLIP: FLICE inhibitory protein
IAP: suppressors of apoptosis
MTX: Methotrexate
TRAIL: TNF-related apoptosis-inducing ligand
XIAP: X-linked inhibitor of apoptosis
