# Supplementary material for: Cell cycle-arrested tumor cells exhibit increased sensitivity towards TRAIL-induced apoptosis
Source: Cell Death Dis. 2013 Jun 6;4(6):e661–. doi: 10.1038/cddis.2013.179 (PMC3698546; doi:10.1038/cddis.2013.179)
Supplement: Supplementary Figures [file cddis2013179x1.ppt]

## Slide 1
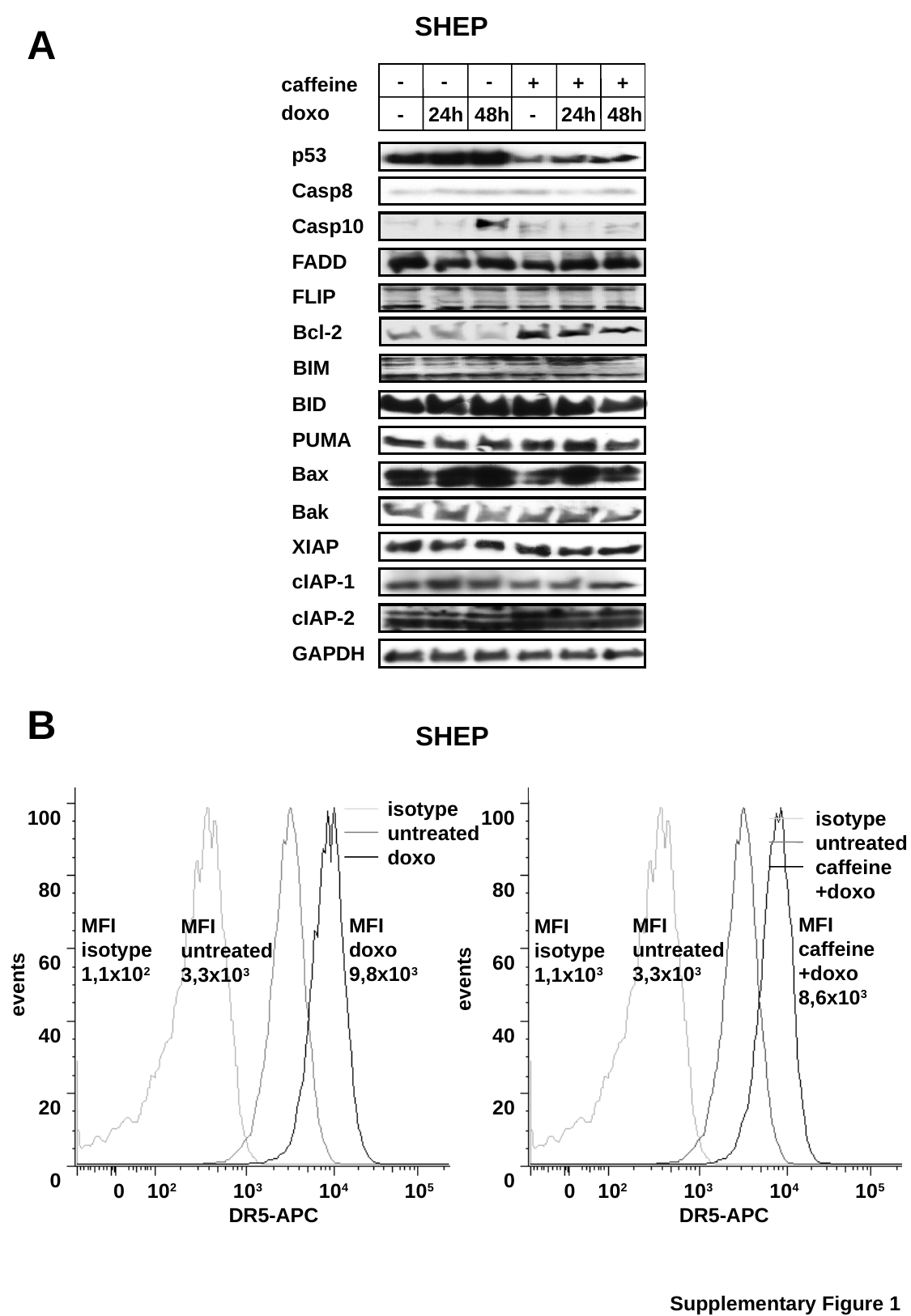

SHEP
A
B
caffeine
doxo
-
-
-
+
+
+
-
24h 48h
-
24h 48h
p53
Casp8
Casp10
FADD
FLIP
Bcl-2
BIM
BID
PUMA
Bax
Bak
XIAP
cIAP-1
cIAP-2
GAPDH
SHEP
100
80
60
40
20
0
100
80
60
40
20
0
isotype
untreated
doxo
isotype
untreated
caffeine
+doxo
MFI
caffeine
+doxo
8,6x10^3^
MFI
isotype
1,1x10^2^
MFI
doxo
9,8x10^3^
MFI
untreated
3,3x10^3^
MFI
isotype
1,1x10^3^
MFI
untreated
3,3x10^3^
events
events
0 102 103 104 105
DR5-APC
0 102 103 104 105
DR5-APC
Supplementary Figure 1

## Slide 2
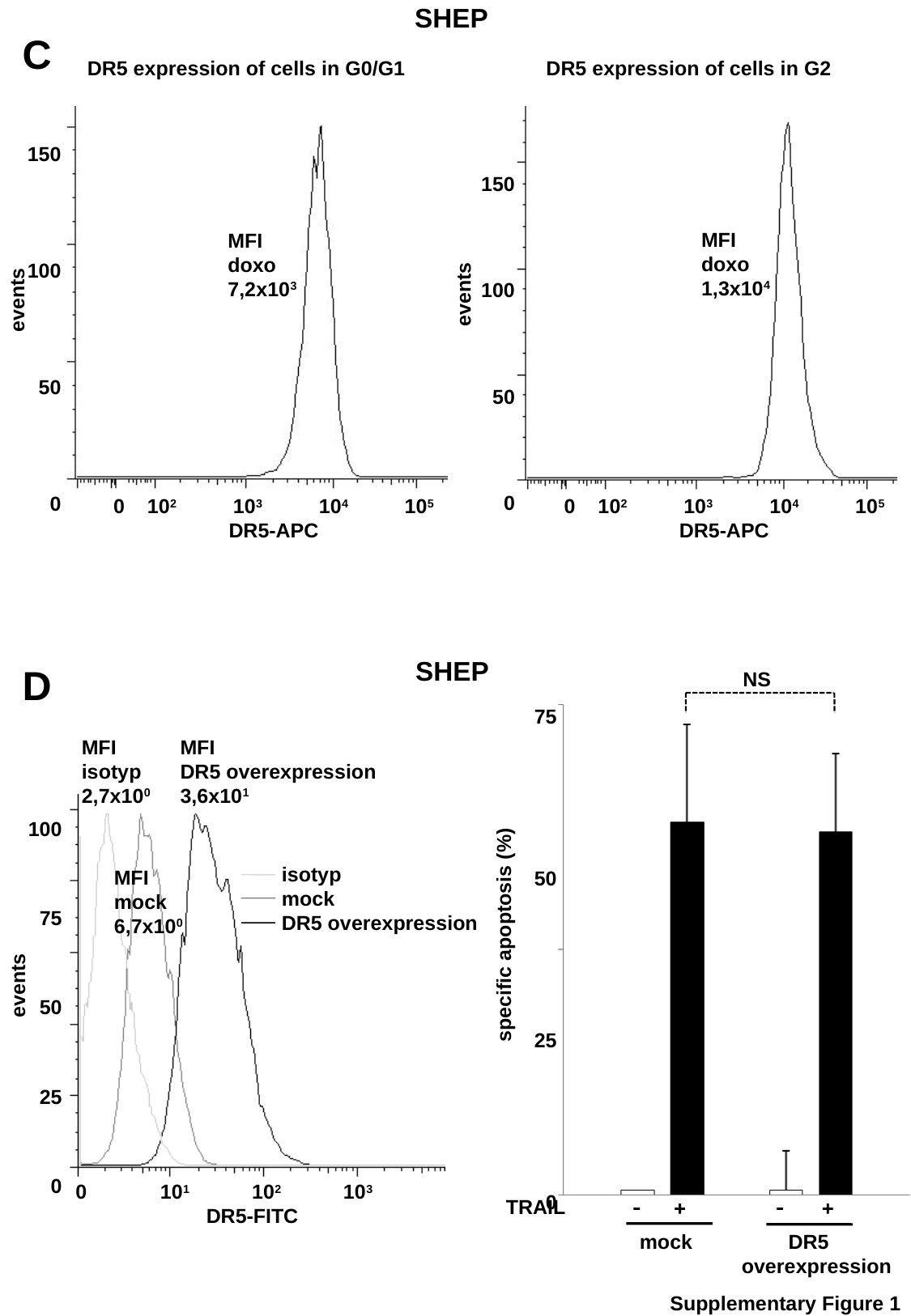

SHEP
C
D
150
100
50
0
DR5 expression of cells in G0/G1 DR5 expression of cells in G2
150
100
50
0
MFI
doxo
1,3x10^4^
MFI
doxo
7,2x10^3^
events
events
0 102 103 104 105
DR5-APC
0 102 103 104 105
DR5-APC
75
50
25
0
SHEP
NS
MFI
isotyp
2,7x100
MFI
DR5 overexpression
3,6x101
100
75
50
25
0
isotyp
mock
DR5 overexpression
MFI
mock
6,7x100
specific apoptosis (%)
events
0 101 102 103
DR5-FITC
TRAIL − + − +
mock DR5
 overexpression
Supplementary Figure 1

## Slide 3
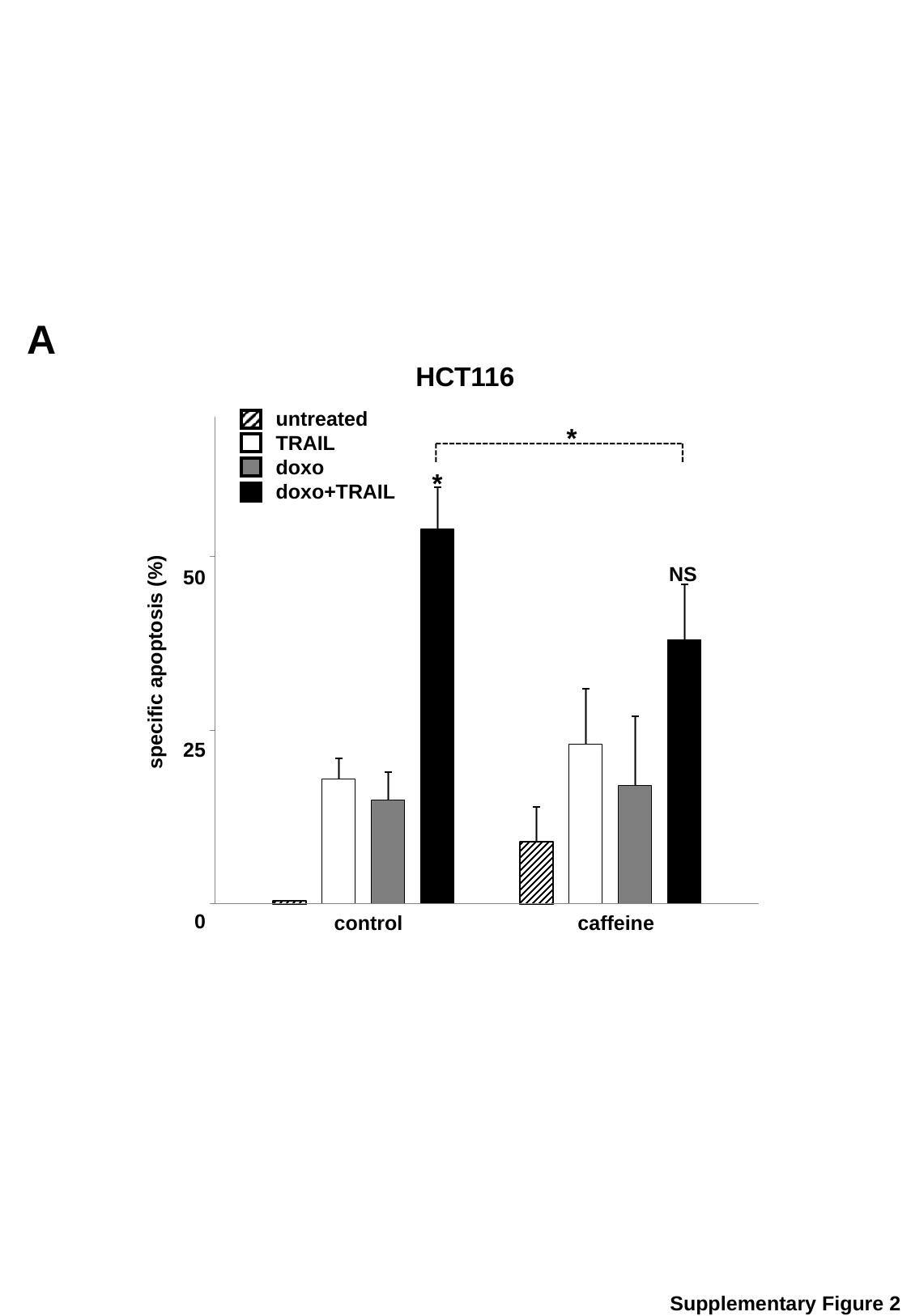

A
untreated
TRAIL
doxo
doxo+TRAIL
*
50
25
0
NS
specific apoptosis (%)
 control caffeine
HCT116
*
Supplementary Figure 2

## Slide 4
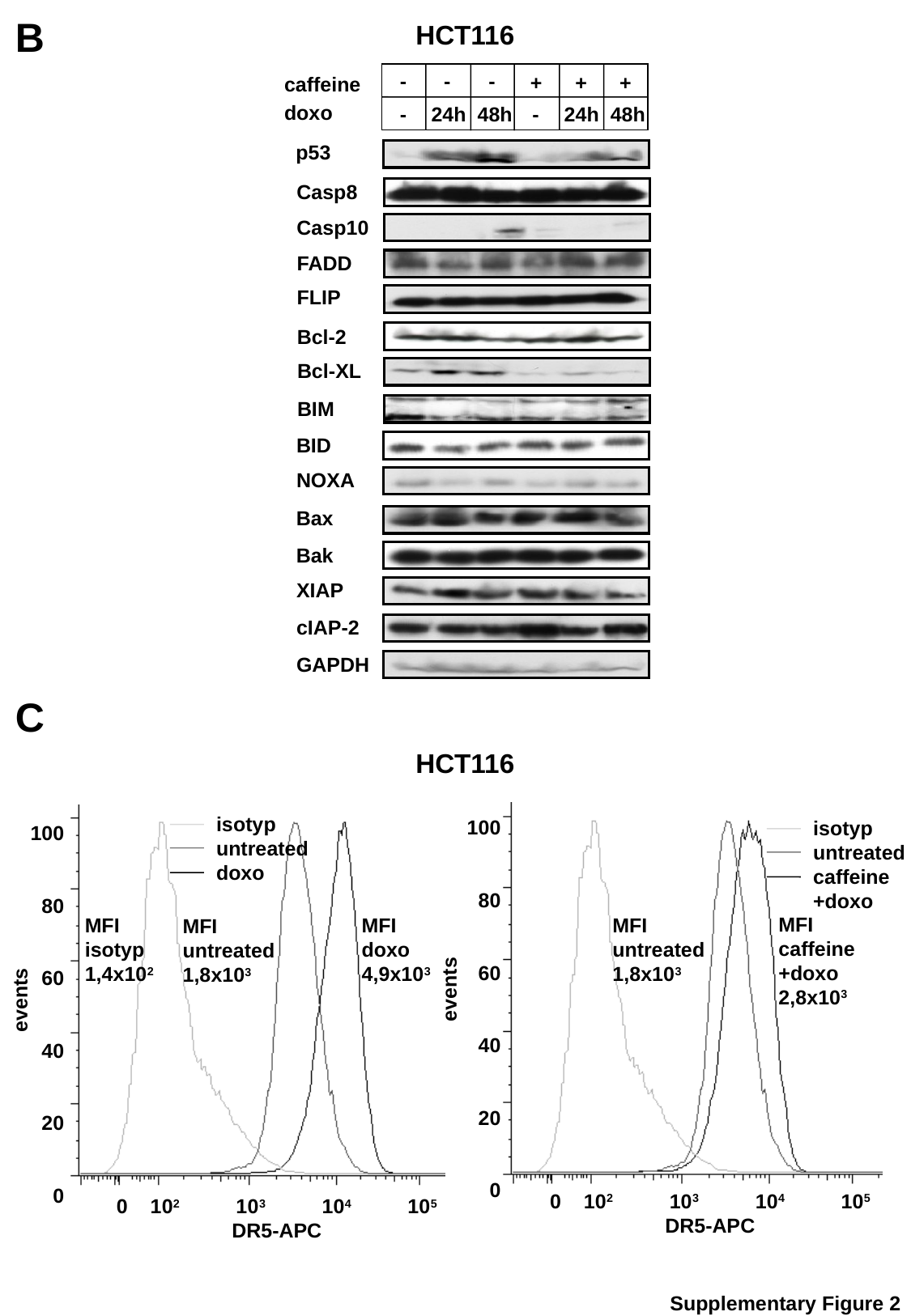

B
C
HCT116
caffeine
doxo
-
-
-
+
+
+
-
24h 48h
-
24h 48h
p53
Casp8
Casp10
FADD
FLIP
Bcl-2
Bcl-XL
BIM
BID
NOXA
Bax
Bak
XIAP
cIAP-2
GAPDH
HCT116
100
80
60
40
20
0
100
80
60
40
20
0
isotyp
untreated
doxo
events
0 102 103 104 105
DR5-APC
isotyp
untreated
caffeine
+doxo
MFI
caffeine
+doxo
2,8x10^3^
MFI
isotyp
1,4x10^2^
MFI
doxo
4,9x10^3^
MFI
untreated
1,8x10^3^
MFI
untreated
1,8x10^3^
events
0 102 103 104 105
DR5-APC
Supplementary Figure 2

## Slide 5
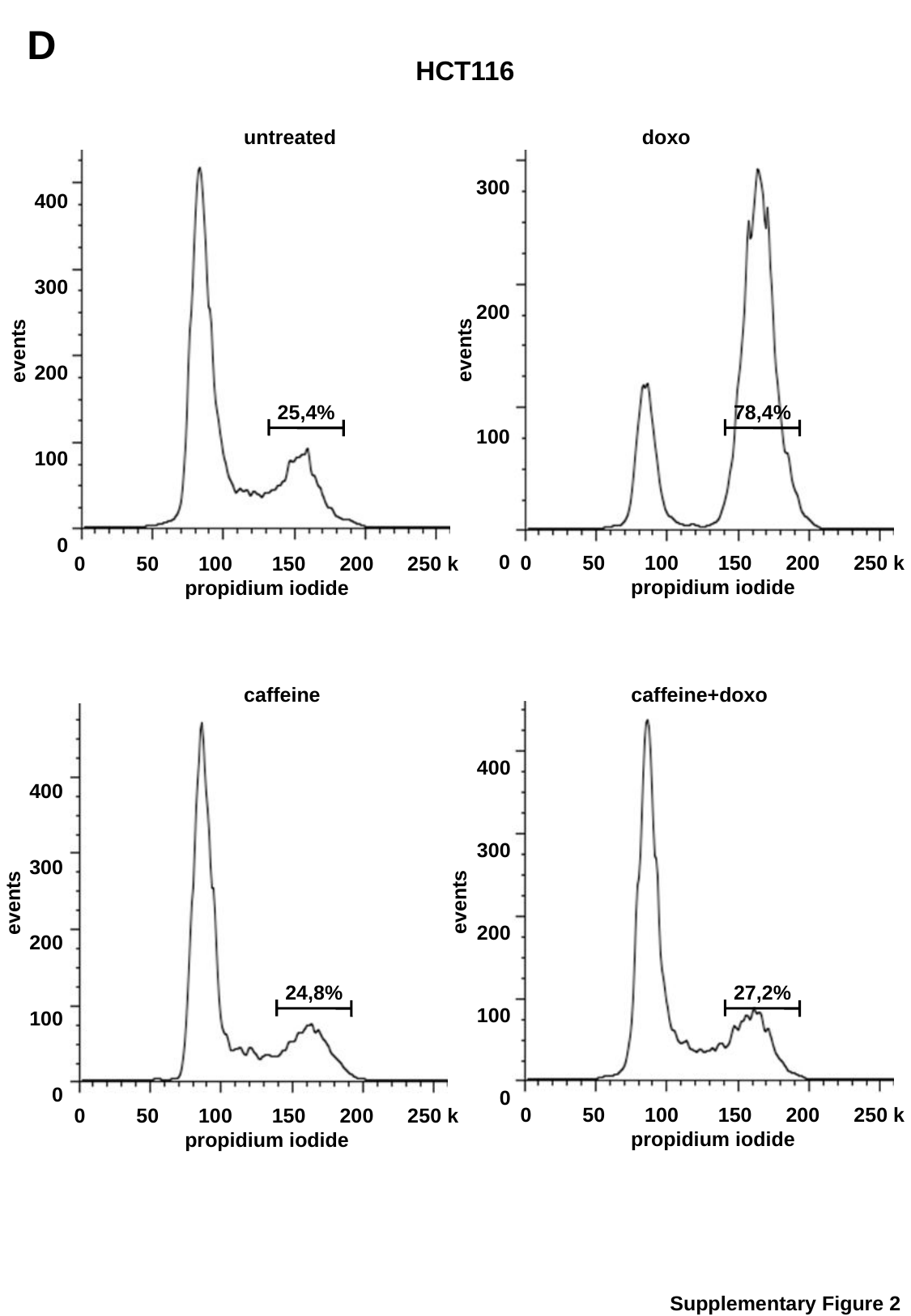

D
HCT116
300
200
100
0
untreated		 	 doxo
caffeine caffeine+doxo
400
300
200
100
0
events
events
25,4%
78,4%
0 50 100 150 200 250 k
propidium iodide
0 50 100 150 200 250 k
propidium iodide
400
300
200
100
0
400
300
200
100
0
events
events
24,8%
27,2%
0 50 100 150 200 250 k
propidium iodide
0 50 100 150 200 250 k
propidium iodide
Supplementary Figure 2

## Slide 6
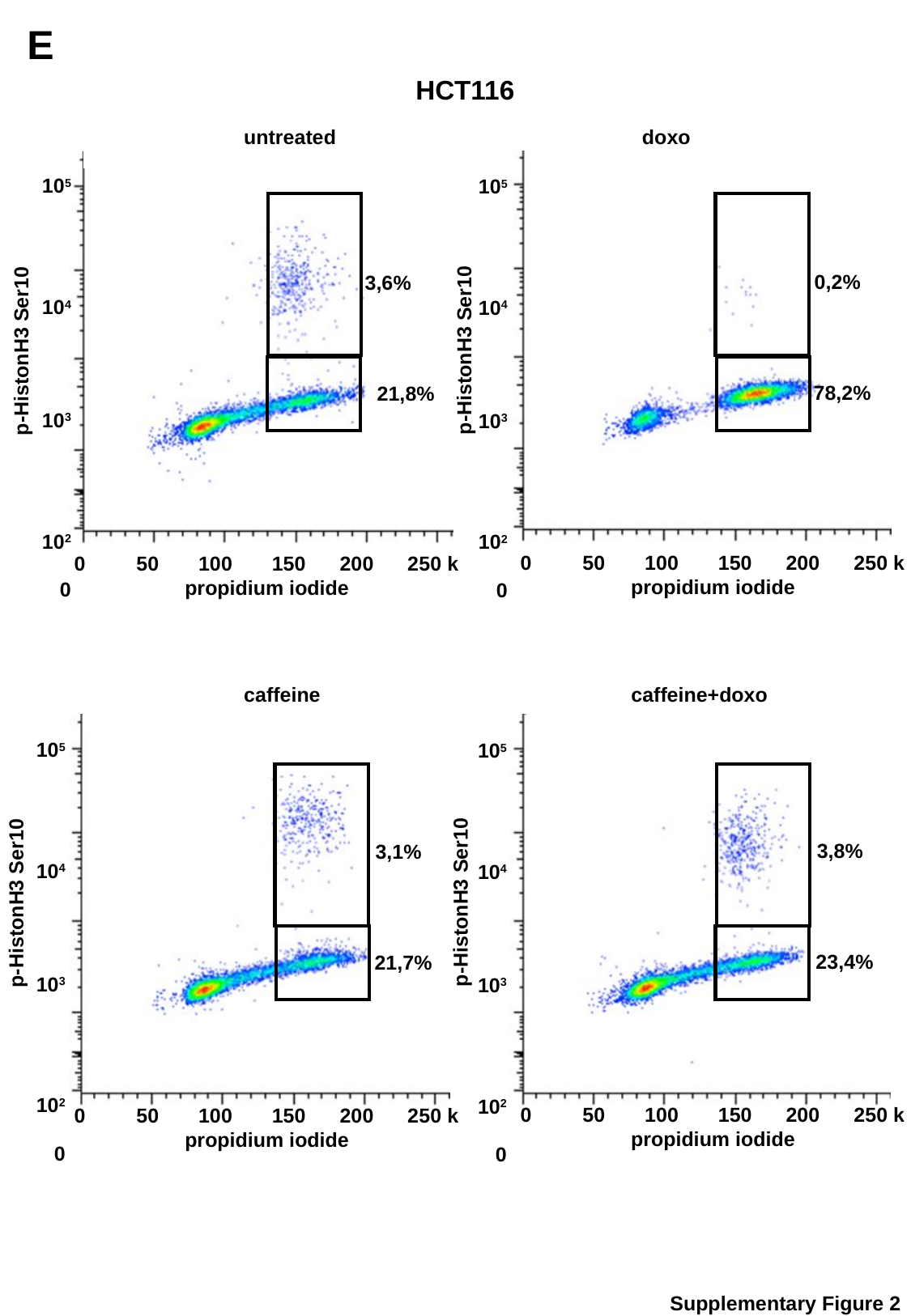

E
HCT116
untreated		 	 doxo
caffeine caffeine+doxo
105
104
103
102
0
105
104
103
102
0
0,2%
3,6%
p-HistonH3 Ser10
p-HistonH3 Ser10
78,2%
21,8%
0 50 100 150 200 250 k
propidium iodide
0 50 100 150 200 250 k
propidium iodide
105
104
103
102
0
105
104
103
102
0
3,8%
3,1%
p-HistonH3 Ser10
p-HistonH3 Ser10
23,4%
21,7%
0 50 100 150 200 250 k
propidium iodide
0 50 100 150 200 250 k
propidium iodide
Supplementary Figure 2

## Slide 7
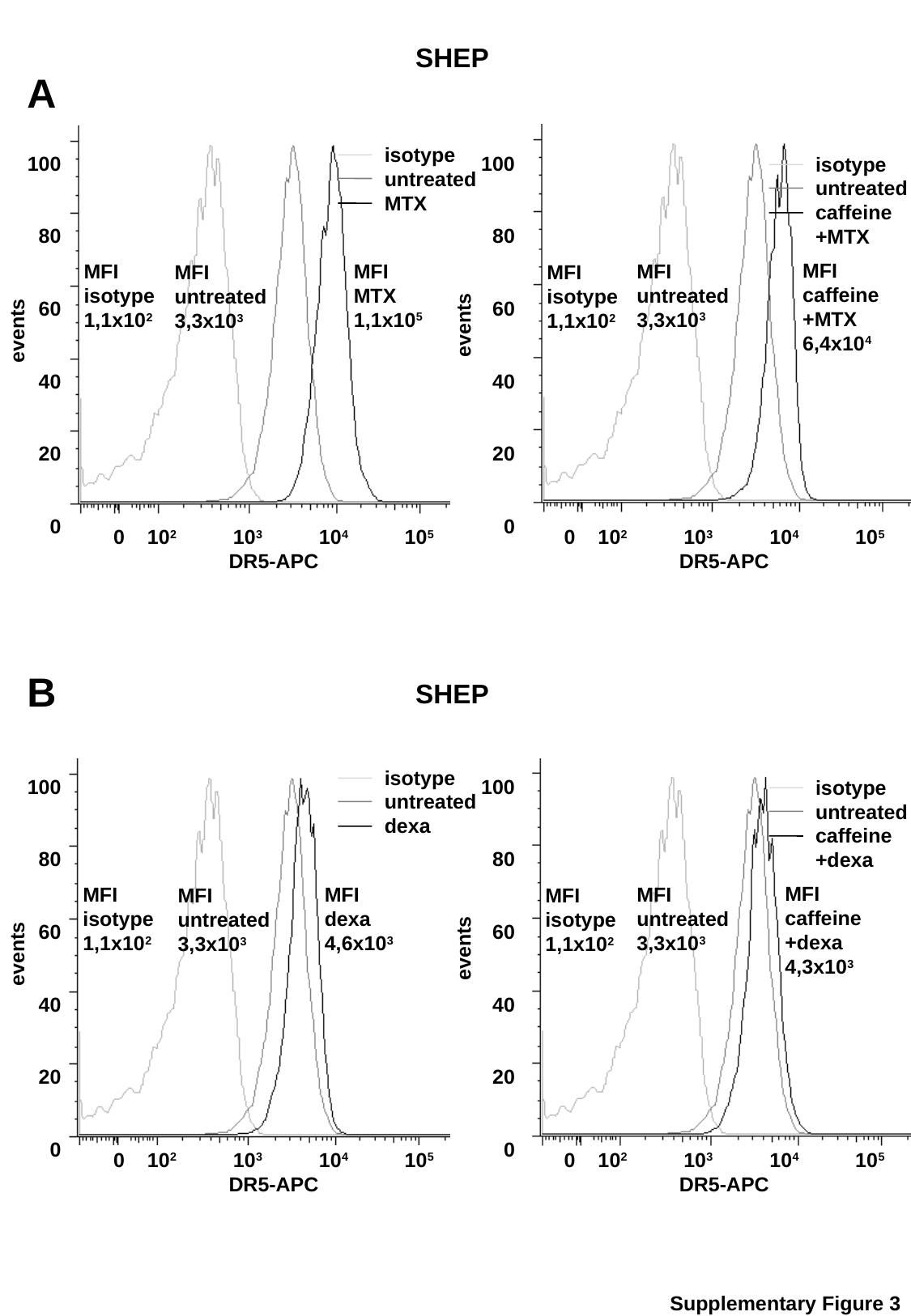

SHEP
A
B
100
80
60
40
20
0
100
80
60
40
20
0
isotype
untreated
MTX
isotype
untreated
caffeine
+MTX
MFI
caffeine
+MTX
6,4x10^4^
MFI
isotype
1,1x10^2^
MFI
MTX
1,1x10^5^
MFI
untreated
3,3x10^3^
MFI
untreated
3,3x10^3^
MFI
isotype
1,1x10^2^
events
events
0 102 103 104 105
DR5-APC
0 102 103 104 105
DR5-APC
SHEP
100
80
60
40
20
0
100
80
60
40
20
0
isotype
untreated
dexa
isotype
untreated
caffeine
+dexa
MFI
caffeine
+dexa
4,3x10^3^
MFI
isotype
1,1x10^2^
MFI
dexa
4,6x10^3^
MFI
untreated
3,3x10^3^
MFI
untreated
3,3x10^3^
MFI
isotype
1,1x10^2^
events
events
0 102 103 104 105
DR5-APC
0 102 103 104 105
DR5-APC
Supplementary Figure 3

## Slide 8
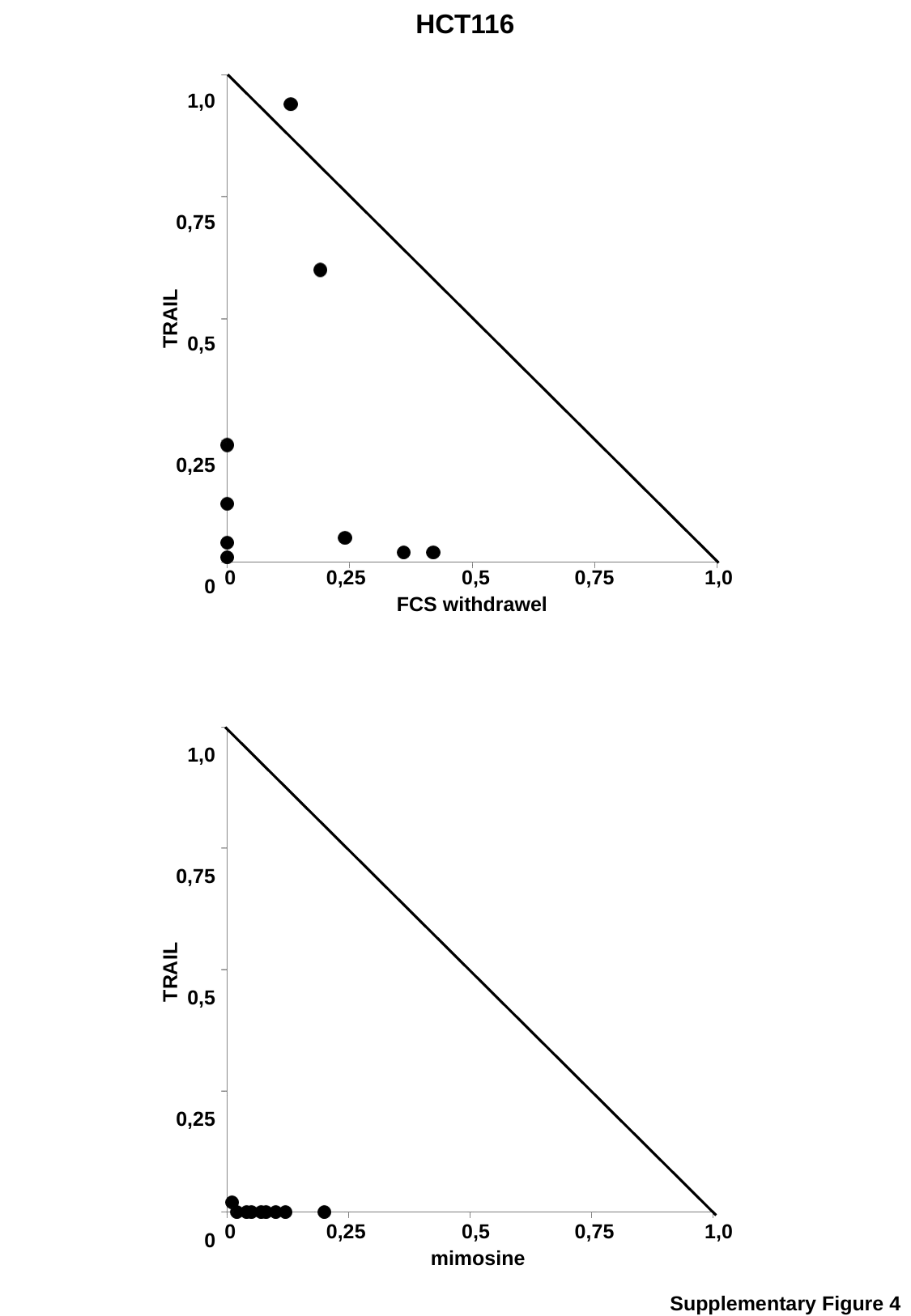

1,0
0,75
0,5
0,25
0
HCT116
TRAIL
0 0,25 0,5 0,75 1,0
FCS withdrawel
1,0
0,75
0,5
0,25
0
TRAIL
0 0,25 0,5 0,75 1,0
mimosine
Supplementary Figure 4

## Slide 9
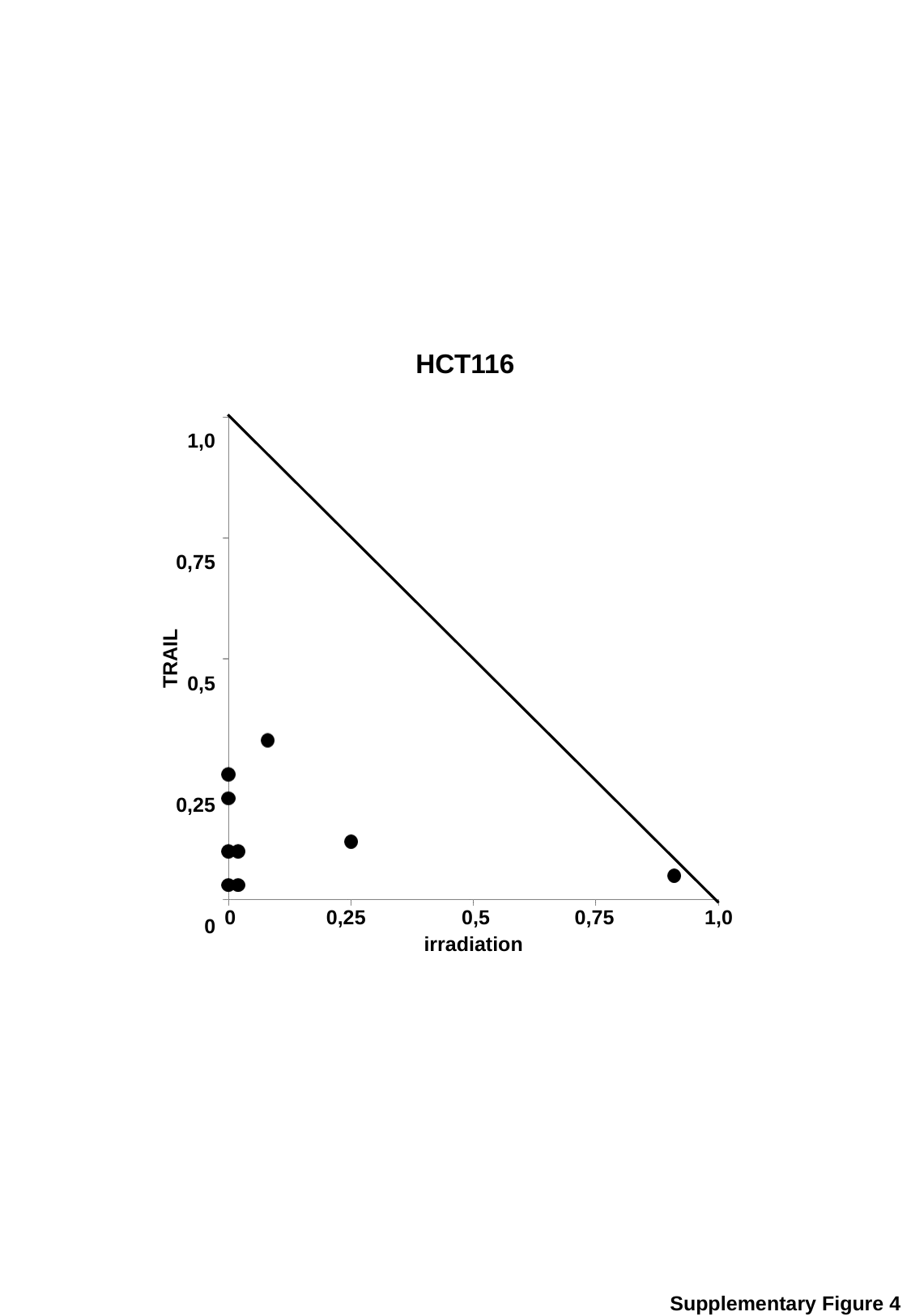

1,0
0,75
0,5
0,25
0
HCT116
TRAIL
0 0,25 0,5 0,75 1,0
irradiation
Supplementary Figure 4

## Slide 10
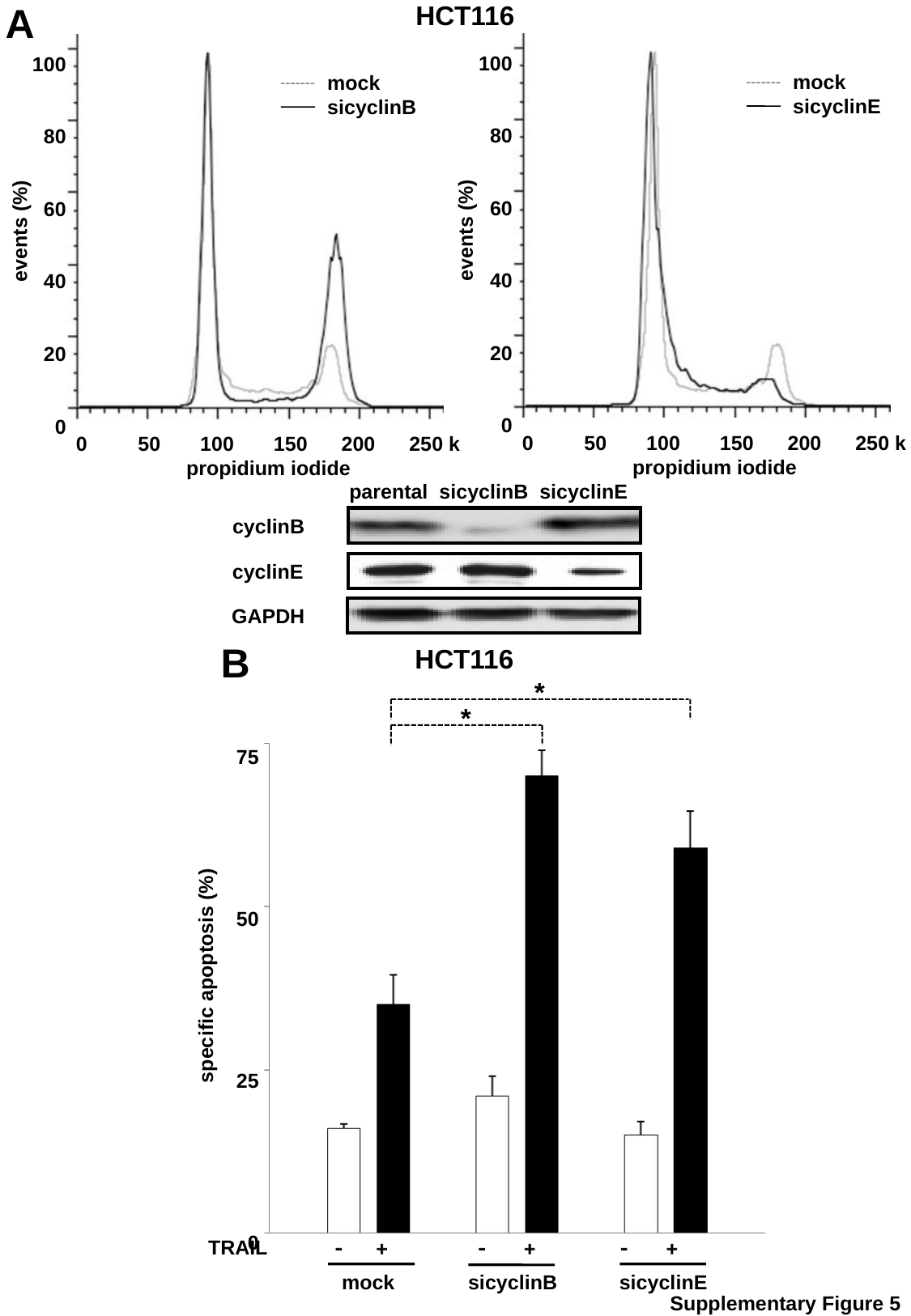

A
 B
HCT116
100
80
60
40
20
0
100
80
60
40
20
0
mock
sicyclinE
mock
sicyclinB
events (%)
events (%)
0 50 100 150 200 250 k
propidium iodide
0 50 100 150 200 250 k
propidium iodide
parental sicyclinB sicyclinE
cyclinB
cyclinE
GAPDH
HCT116
*
75
50
25
0
*
specific apoptosis (%)
TRAIL − + − + − +
mock sicyclinB sicyclinE
Supplementary Figure 5

## Slide 11
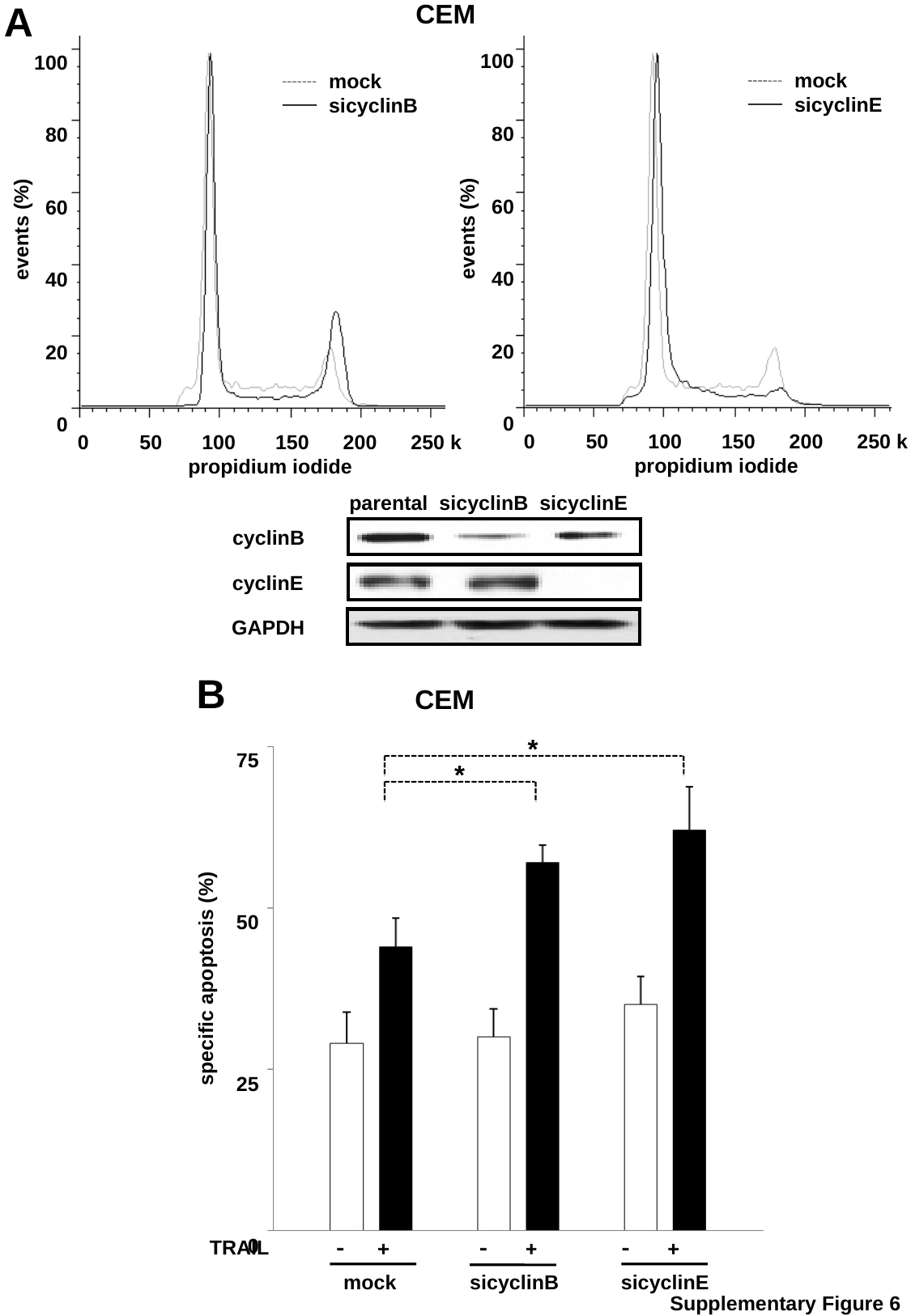

A
 B
CEM
100
80
60
40
20
0
100
80
60
40
20
0
mock
sicyclinE
mock
sicyclinB
events (%)
events (%)
0 50 100 150 200 250 k
propidium iodide
0 50 100 150 200 250 k
propidium iodide
parental sicyclinB sicyclinE
cyclinB
cyclinE
GAPDH
CEM
75
50
25
0
*
*
specific apoptosis (%)
TRAIL − + − + − +
mock sicyclinB sicyclinE
Supplementary Figure 6

## Slide 12
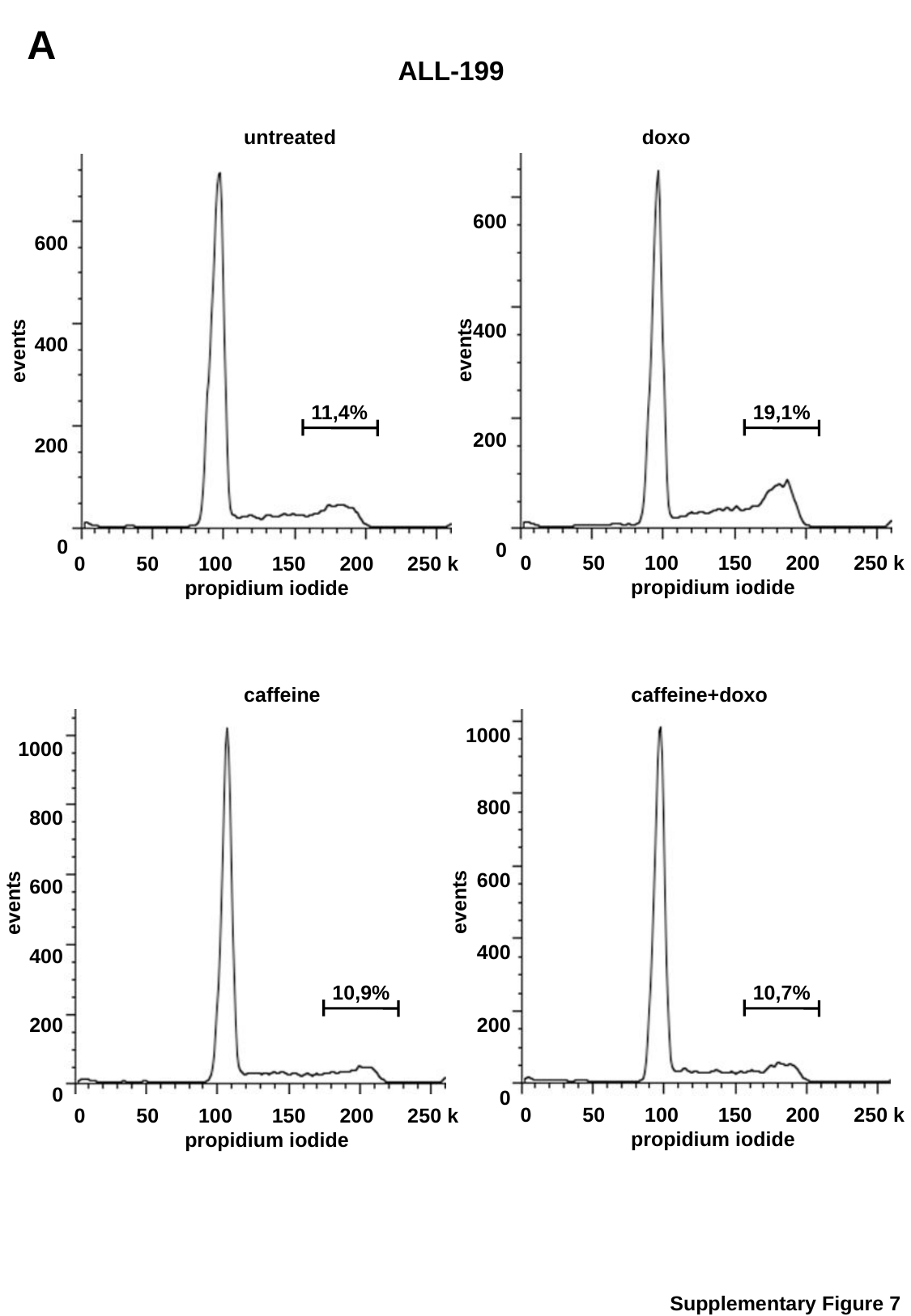

A
ALL-199
600
400
200
0
untreated		 	 doxo
caffeine caffeine+doxo
600
400
200
0
events
events
11,4%
19,1%
0 50 100 150 200 250 k
propidium iodide
0 50 100 150 200 250 k
propidium iodide
1000
800
600
400
200
0
1000
800
600
400
200
0
events
events
10,9%
10,7%
0 50 100 150 200 250 k
propidium iodide
0 50 100 150 200 250 k
propidium iodide
Supplementary Figure 7

## Slide 13
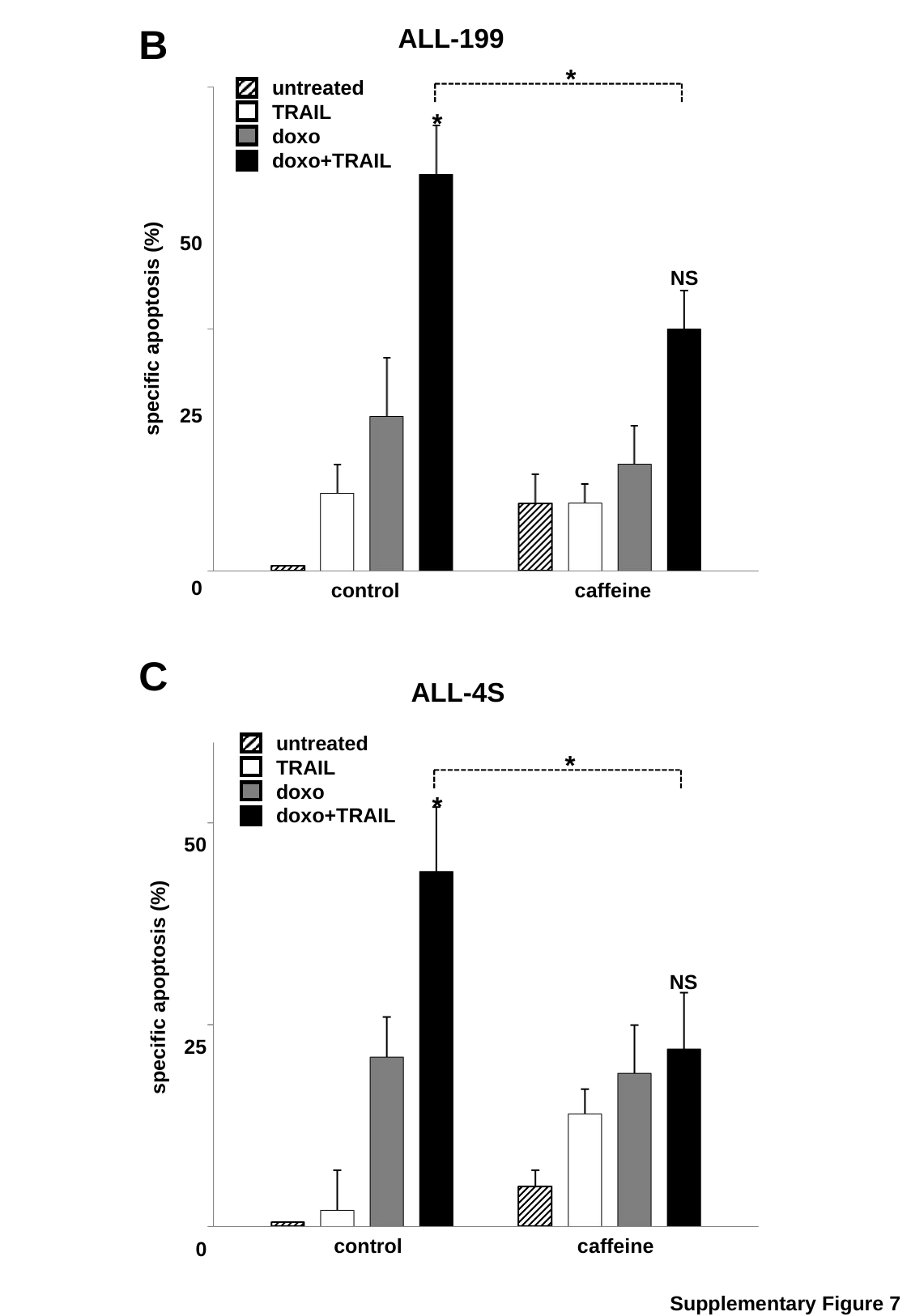

B
C
ALL-199
*
untreated
TRAIL
doxo
doxo+TRAIL
*
50
25
0
NS
specific apoptosis (%)
 control caffeine
ALL-4S
untreated
TRAIL
doxo
doxo+TRAIL
*
50
25
0
*
NS
specific apoptosis (%)
 control caffeine
Supplementary Figure 7

## Slide 14
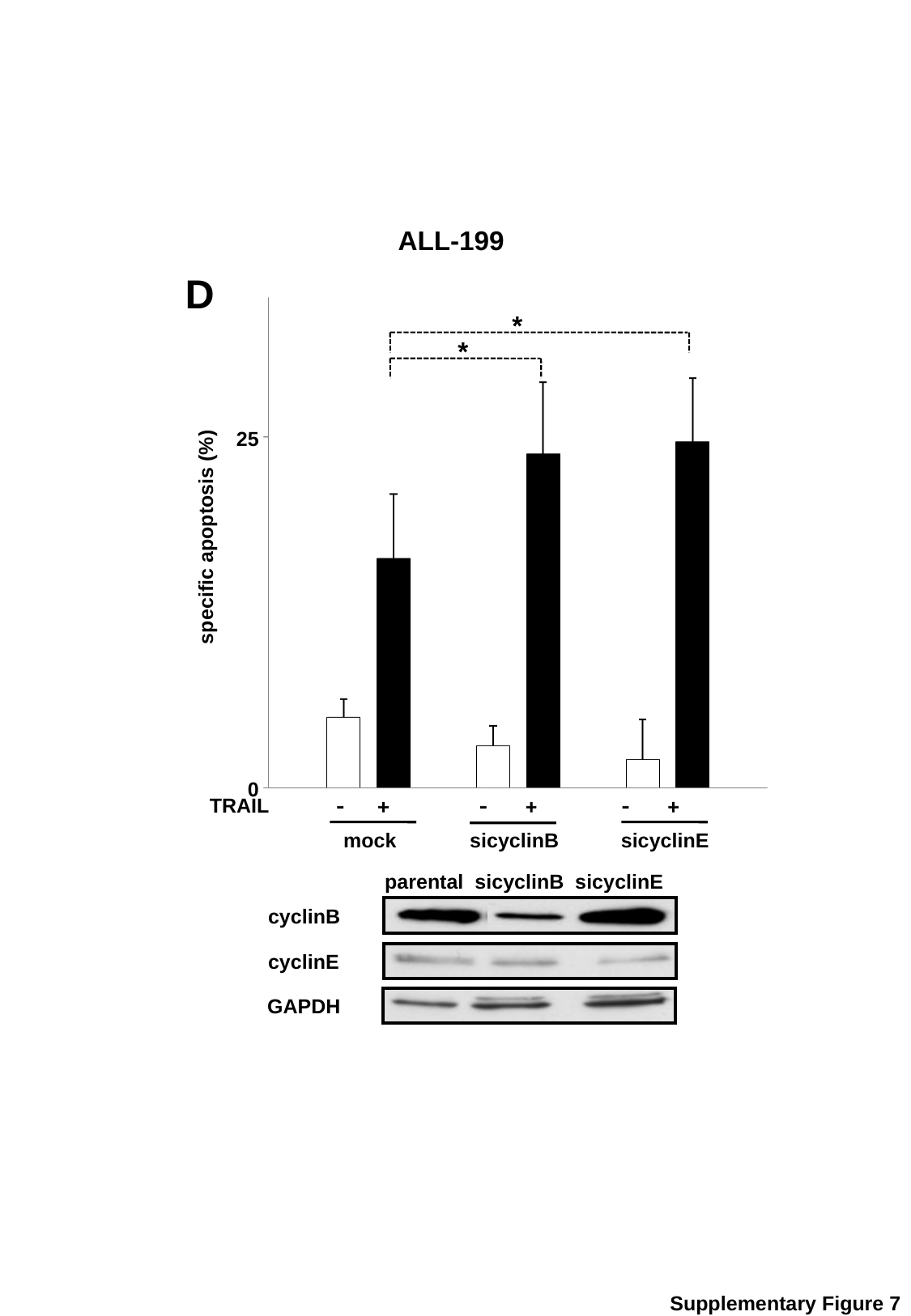

ALL-199
D
*
*
25
0
specific apoptosis (%)
TRAIL − + − + − +
mock sicyclinB sicyclinE
parental sicyclinB sicyclinE
cyclinB
cyclinE
GAPDH
Supplementary Figure 7

## Slide 15
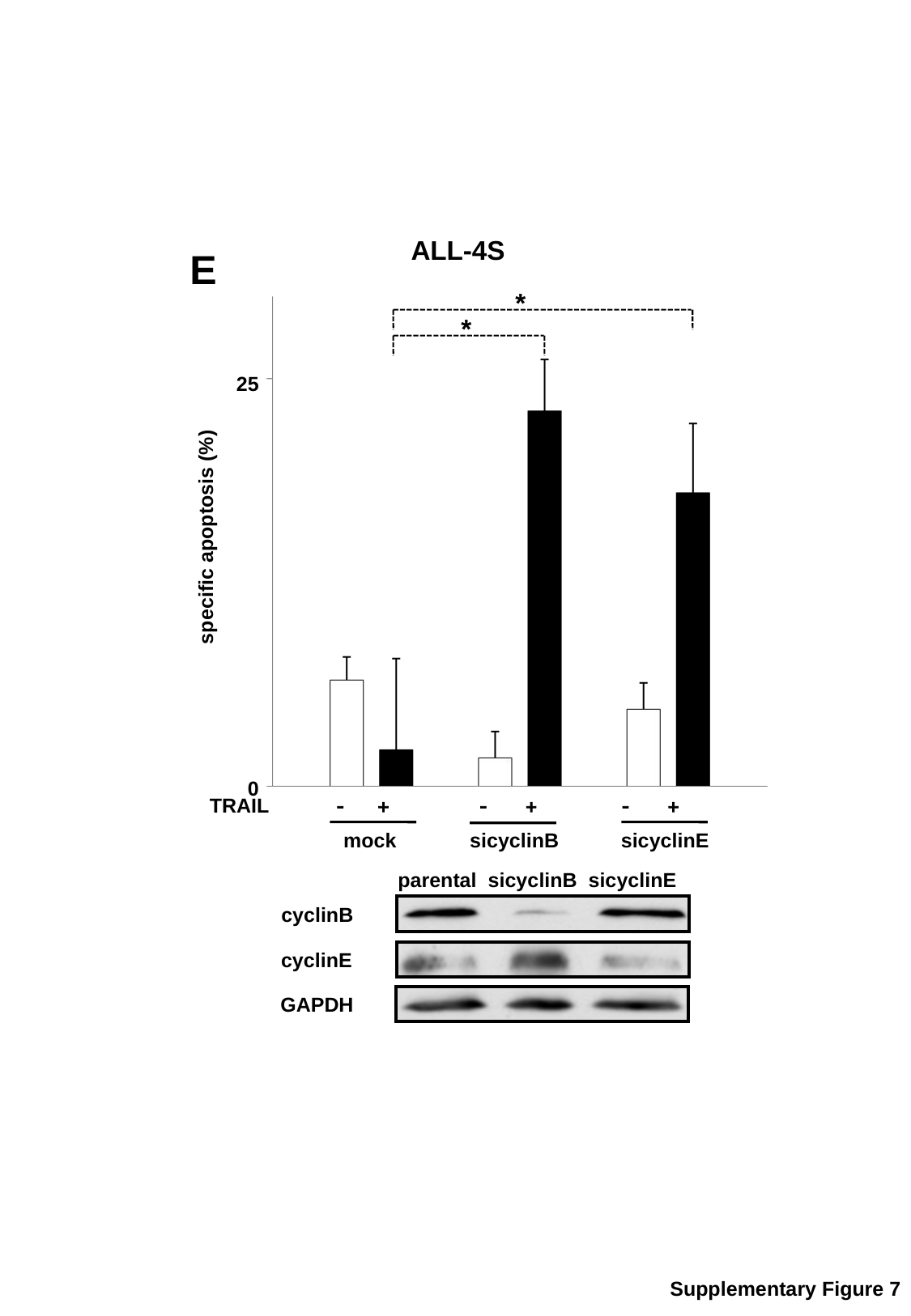

ALL-4S
E
*
*
25
0
specific apoptosis (%)
TRAIL − + − + − +
mock sicyclinB sicyclinE
parental sicyclinB sicyclinE
cyclinB
cyclinE
GAPDH
Supplementary Figure 7
