# Supplementary material for: Cell cycle-arrested tumor cells exhibit increased sensitivity towards TRAIL-induced apoptosis
Source: Cell Death Dis. 2013 Jun 6;4(6):e661–. doi: 10.1038/cddis.2013.179 (PMC3698546; doi:10.1038/cddis.2013.179)
Supplement: Supplementary Figures Legends [file cddis2013179x2.doc]

**Titles and legends to Supplementary Figures**

**Supplementary Figure S1 Synergistic activity of doxo and TRAIL dependent on p53 in SHEP cells**

A,B) SHEP cells treated as in Figure 1 were analysed for the expression level of apoptosis proteins from whole cell lysates (A) or for TRAIL receptor-2 expression (B) as described in Materials and Methods. Isotype=isotype control, control=death receptor expression of unstimulated cells. GAPDH served as loading control.

C) **SHEP cells from Figure 1A treated with doxo were separated for cell cycle distribution in G0/G1 (left panel) and G2 (right panel). TRAIL receptor-2 expression was analysed as in Supplementary Figure 1B.**

**D) TRAIL receptor-2 was overexpressed in SHEP cells. 24 hours later, overexpression of TRAIL receptor-2 was verified by DR5 surface staining (left panel). After 48 hours, mock and TRAIL receptor-2 overexpressing SHEP cells were stimulated with TRAIL (100 ng/ml) for 12 hours.**

Presentation and analysis of the data and statistical analysis were performed as in Figure 1.

**Supplementary Figure S2 Synergistic activity of doxo and TRAIL dependent on p53 in HCT116 cells**

1. Parental HCT116 cells with p53 wild-type status were stimulated and analysed as in Figure 1A.

B,C) HCT116 cells from Supplementary Figure S2A were analysed as in Supplementary Figure 1A and B.

D,E) Parental HCT116 cells from Supplementary Figure S2A were analysed for the cell cycle distribution as in Figure 1C (D) and D (E).

Determination of apoptosis induction, presentation and analysis of the data and statistical analysis were performed as in Figure 1 and 2.

**Supplementary Figure S3 Impact of MTX and dexa on TRAIL receptor-2 expression**

A,B) SHEP cells treated with MTX (A) or dexa (B) as in Figure 2 were analysed for TRAIL receptor-2 expression as in Supplementary Figure 1B.

**Supplementary Figure S4 Biochemical cell cycle arrest of HCT116 cells sensitizes for TRAIL**

HCT116 cells were treated and analysed as in Figure 3B. Irradiation was performed with 10, 30 and 60Gy, TRAIL was added with 10, 30, 100 ng/ml.

Determination of apoptosis induction, presentation and analysis of the data were performed as in Figure 3B.

**Supplementary Figure S5 Impact of molecular cell cycle arrest on TRAIL sensitivity in HCT116 cells**

A,B) HCT116 cells repetitively transfected with siRNA against cyclinB or cyclinE as described in Materials and Methods were analysed for the cell cycle distribution (A) and TRAIL sensitivity (B) as in Figure 4A and F.

Determination of apoptosis induction, presentation and analysis of the data and statistical analysis were performed as in Figure 4.

**Supplementary Figure S6 Impact of molecular cell cycle arrest on TRAIL sensitivity in CEM cells**

A,B) Parental CEM cells were repetitively transfected with siRNA against cyclinB or cyclinE as described in Materials and Methods. Cells were subjected to cell cycle analysis (A) or stimulated with TRAIL (30 ng/ml) for another 24 hours (B) as in Figure 4A and F.

Determination of apoptosis induction, presentation and analysis of the data and statistical analysis were performed as in Figure 4.

**Supplementary Figure S7 The contribution of cell cycle arrest for efficient TRAIL apoptosis induction in xenografted ALL cells**

A,B) Xenografted pre-B ALL-199 cells were treated and analysed as in Figure 5A and B.

C) Xenografted T ALL-4S cells were stimulated with caffeine (200 µg/ml), doxo (10 ng/ml) and TRAIL (10 ng/ml) as in Figure 5B.

D,E) ALL-199 (D) and ALL-4S (E) cells transiently transfected with siRNA against cyclinB, cyclinE or a mock sequence as in Figure 6 were stimulated with TRAIL (10 ng/ml) for another 24 hours. In Figure D the order of samples within the Western Blot was rearranged without any further manipulation for the ease of reading.

Determination of cell cycle distribution and apoptosis induction, presentation and analysis of the data and statistical analysis were performed as in Figure 5 and 6.
