# Supplementary material for: Cell cycle-arrested tumor cells exhibit increased sensitivity towards TRAIL-induced apoptosis
Source: Cell Death Dis. 2013 Jun 6;4(6):e661–. doi: 10.1038/cddis.2013.179 (PMC3698546; doi:10.1038/cddis.2013.179)
Supplement: Supplementary Table 1 [file cddis2013179x3.doc]

**Supplementary Table 1.** Cell cycle distribution in HCT116 cells after cell cycle inhibition

| Cell cycle distribution | G0 | G1 | G2 | M |
| --- | --- | --- | --- | --- |
| control | 3,5±0,2% | 54,9±0,4% | 26,3±0,7% | 3,1±0,1% |
| 0% FCS | 42,1±2,1% | 30,4±1,8% | 18,7±0,1% | 0,7±0,3% |
| mimosine | 2,2±0,7% | 68,1±0,7% | 17,7±0,1% | 0,8±0,1% |
| irradiation | 0,8±0,4% | 15,3±7,5% | 69,6±10,5% | 1,1±0,1% |

HCT116 cells were treated by withdrawal of FCS (0%FCS), with mimosine (100µM) or irradiated (30Gy) for 24 hours. Cell cycle distribution was analyzed with propidium iodide in combination with cyclinD1 staining to discriminate G0 and G1 phase and with p-Histone-H3 staining to separate arrest in G2 and M-phase. Data are presented as mean ± SEM.
